# Associations between Dietary Patterns and Incident Colorectal Cancer in 114,443 Individuals from the UK Biobank: A Prospective Cohort Study

**DOI:** 10.1158/1055-9965.EPI-24-0048

**Published:** 2024-08-19

**Authors:** Samuel L. Skulsky, Dimitrios A. Koutoukidis, Jennifer L. Carter, Carmen Piernas, Susan A. Jebb, Min Gao, Nerys M. Astbury

**Affiliations:** 1 Nuffield Department of Population Health, University of Oxford, Oxford, United Kingdom.; 2 Nuffield Department of Primary Care Health Sciences, University of Oxford, Oxford, United Kingdom.; 3 Department of Biochemistry and Molecular Biology II, Faculty of Pharmacy; Centre for Biomedical Research; Institute of Nutrition and Food Technology; Biosanitary Research Institute ibs.Granada; University of Granada, Spain; 4 NIHR Oxford Biomedical Research Centre, Oxford, United Kingdom.; 5 NIHR Oxford Health Biomedical Research Centre, Oxford, United Kingdom.

## Abstract

**Background:**

Diet–disease association studies increasingly use dietary patterns (DP) to account for the complexity of the exposure. We assessed if a DP associated with type 2 diabetes mellitus, cardiovascular disease, and all-cause mortality is also associated with colorectal cancer.

**Methods:**

We used reduced rank regression on 24-hour recall data to identify DPs, explaining the maximum variation in four nutrient-response variables: energy density, saturated fatty acids, free sugars, and fiber density. Cox proportional hazards models examined prospective associations between DP adherence (coded in a continuous scale as *z*-scores as well as in quintiles) and incident colorectal cancer. Subgroup analyses were conducted for tumor site, age, and sex.

**Results:**

After exclusions, 1,089 colorectal cancer cases occurred in 114,443 participants over a median follow-up of 8.0 years. DP1 was characterized by increased intake of chocolate and confectionery; butter; low-fiber bread; red and processed meats; and alcohol, as well as low intake of fruits, vegetables, and high-fiber cereals. After accounting for confounders, including body mass, there were positive linear associations between DP1 and incident overall colorectal cancer (HR of quintile 5 vs. 1, 1.34; 95% confidence interval, 1.16–1.53, *P*_trend_ = 0.005) and rectal cancer (HR of quintile 5 vs. 1, 1.58; 95% confidence interval, 1.27–1.96, *P*_trend_ = 0.009) but not for proximal or distal colon cancers. No DP2–colorectal cancer association was observed.

**Conclusions:**

A DP previously associated with cardiometabolic disease is also associated with incident colorectal cancer, especially rectal cancers.

**Impact:**

These consistent associations of particular food groups with both cardiometabolic disease and this diet-related cancer strengthen the evidence base for holistic population dietary guidelines to prevent ill-health.

## Introduction

Globally, colorectal cancer is the second most diagnosed cancer in women and third in men (RRID: SCR_025451). The incidence of colorectal cancer varies widely by geographic region, with higher rates generally observed in developed Western nations compared with Sub-Saharan Africa (excluding South Africa) and Southern Asia (1). Although the incidence rate has declined in some countries (2), overall global incidence is gradually increasing (1). Additionally, surveillance studies have identified increased incidence rates in younger individuals; in the United States, the proportion of new colorectal cancer cases for adults less than 55 years increased by almost 50% from 1995 to 2019 (2). This trend is mirrored in global populations (1, 3, 4), with a predicted increase in crude overall incidence for individuals less than 50 years of 1% to 3% annually (1).

Suboptimal lifestyle patterns—including diet—have been proposed as contributors to the rapid increase in colorectal cancer in regions such as Asia, Eastern Europe, and South America. Increasing obesity rates and shifts in developing regions toward “Western” dietary patterns (DP) are thought to be associated with incident colorectal cancer (5). Although it is variably defined in the literature, a Western DP is generally characterized by higher intakes of red and processed meats, added free sugars, refined grains, saturated fats, and low-fiber foods and poor intakes of fruits and vegetables (6, 7).

Such DPs have been associated with unfavorable alteration in the gut microbiota composition, contributing to an increased risk of weight gain and obesity (8), an established risk factor for colorectal cancer. Furthermore, specific food items have been associated with colorectal cancer. For example, meta-analysis of 10 prospective cohort studies found that for every 50 g/day of processed meats and 100 g/day of red meat eaten, the relative risk of colorectal cancer increases by ∼16% and 12%, respectively (9). In contrast, other dietary components seem to confer protection against colorectal cancer. A meta-analysis of 25 prospective studies found that the incidence rate of colorectal cancer decreased by 10% for every 10 g/day increase in dietary fiber consumption (10). However, not all studies yielded concordant results; a pooled analysis of 13 cohort studies found that the inverse association between dietary fiber and colorectal cancer risk was abrogated after accounting for other food items (11).

Historically, studies have focused on single (or few) nutrients or food items for associations with disease. This approach struggles to account for the synergistic effects of foods consumed and to capture a realistic representation of the complexity of human diets (12). Additionally, the associations of single nutrients with disease may be affected by residual confounding from unmeasured dietary information. Furthermore, translating evidence on single nutrients or food items into achievable public health recommendations poses practical challenges. “Whole-diet” approaches, examining the associations of DPs (ref. 13) with disease, can address these challenges (6).

A 2017 review of 49 studies examined the association of DPs derived by different methodologies with colorectal cancer (6). From this review emerged the broad theme of a “healthful” eating pattern, characterized by high consumption of fish; milk; whole grains, nuts, and legumes; and fruits and vegetables. In contrast, a “unhealthful/Western” pattern was characterized by high intakes of red and processed meats, refined grains, sugar-sweetened beverages, potatoes, and desserts. Diets mirroring healthful and unhealthful DPs were generally associated with decreased and increased risks of incident colorectal cancer, respectively (6). Associations seem stronger in men than in women and may vary by tumor location (14). However, results have been inconsistent; for example, some studies found stronger positive associations of unhealthful/Western diets with colon cancer (as opposed to rectal cancer; refs. 14–16), whereas other studies found stronger relationships with rectal cancer (as opposed to colon cancer; refs. 7, 17, 18) or no associations with colorectal cancer at all (19–21). The cause of the discrepancy is not fully understood but may be partially influenced by specific study methodologies or populations. For example, Kesse and colleagues (21) found no associations between a Western DP and colorectal cancer, but they only examined women; Williams and colleagues (18) found a stronger association between a Western DP and rectal cancer in Whites but not African Americans.

Reduced rank regression (RRR) is a hybrid approach for DP derivation that uses *a priori* knowledge of nutrients hypothesized to be on the causal pathway for a given disease to examine the association between combinations of food and a disease outcome (22, 23). In contrast to purely data-driven *a posteriori* approaches, this method facilitates focused investigation into diet–disease pathways. Additionally, unlike index-based DPs (e.g., Mediterranean or Dietary Approach to Stop Hypertension diets), it can provide population-specific dietary habits and capture the consumption of culturally relevant food items (24).

In two recent studies of the UK Biobank cohort, Gao and colleagues (23) utilized RRR to derive four DPs explaining variability in energy density, free sugars, saturated fat, and fiber intakes. Two of the four DPs explaining the greatest variation in the response variables were analyzed. The first DP “DP1” was associated with type 2 diabetes mellitus (T2DM), cardiovascular disease (CVD), and all-cause mortality (25). An inspection of DP1’s constituent foods (25) reveals intake levels of multiple food items that have been previously associated with colorectal cancer. Food items (and their intake levels) found in DP1 with strong evidence for increased colorectal cancer risk include increased intakes of red meat, processed meats, and alcohol, as well as decreased intakes of whole-grain/whole-meal foods, high-fiber foods, and low-fat milk (26). Additionally, DP1 is also characterized by intake levels of the following food items with fairly consistent (albeit weaker) evidence for increased colorectal cancer risk: decreased intakes of fish, nonstarchy vegetables, and fruits (26). Furthermore, several food items described in DP1 fall under a typical “Western” diet (7), including chocolate and confectionery; sugar-sweetened beverages; crisps and savory snacks; grain-based desserts; and table sugars and preserves. The second DP “DP2” is characterized by high intakes of sugar-sweetened beverages; fruit juices; table sugars and preserves; chocolate and confectionery; and milk-based and powdered drinks, as well as by low intakes of high-fat cheese, butter, and other animal fat spreads; eggs/egg dishes; red meat; coffee and tea; and processed meats. DP1 and DP2 comprise similar food items but differ in their intakes (25).

Given that DP1 and DP2 are both characterized by food items associated with incident colorectal cancer but vary in the intakes of these food items (e.g., red meat), we hypothesize that the associations of DP1 and DP2 with colorectal cancer may differ. Thus, we aimed to clarify the associations of DP1 and DP2 with incident colorectal cancer in the UK Biobank cohort after controlling for obesity, diabetes, and other confounding factors. Furthermore, we sought to clarify whether the associations are modified by age, sex, or tumor subsite.

## Materials and Methods

### Study design, setting, and participants

The UK Biobank is a prospective cohort study of 502,536 UK men and women ages 37 to 73 years recruited between 2006 and 2010. The UK Biobank protocol can be found online (27). Candidate participants were identified from the National Health Service’s patient registers and invited to an assessment center for evaluation. The National Information Governance Board for Health and Social Care in Wales and England, as well as the Community Health Index Advisory Group in Scotland, approved access to patient records for recruitment purposes. At the assessment centers, participants completed questionnaires, providing clinical, sociodemographic, and lifestyle information. Laboratory, radiographic, and anthropometric measures were also taken. Participant data were linked to hospital and mortality records. At recruitment, all participants provided informed consent for participation and for data linkage. Participants were excluded in the following order: those without or fewer than two validated 24-hour dietary assessments (*n* = 375,680), incident colorectal cancer prior to dietary assessment (*n* = 691), patients withdrawn from the UK Biobank (*n* = 40), previous cancer (excluding nonmelanoma skin cancer, *n* = 8,371), a history of ulcerative colitis (*n* = 845), previous proctocolectomy (surgical removal of the colon and rectum; *n* = 9), or individuals with implausible caloric intakes for their sex (*n* = 1,357). Supplementary Table S1 provides the derivations of exclusions from the UK Biobank datasets. As only a small proportion (*n* = 1,100, 0.95%) of participants were missing data, complete case analysis was performed.

### Study measures

#### Assessment of dietary intake

Dietary intake assessment in the UK Biobank has been previously described (25, 28). Briefly, all participants who provided an email address were invited to complete the Oxford WebQ, an online 24-hour dietary assessment tool designed for large prospective studies (29). It has been validated against biomarkers (30), performs comparably to interviewer-administered 24-hour recalls (31), and demonstrates some degree of reproducibility with two or more completed assessments (32). The Oxford WebQ was administered at baseline and subsequently up to four more times (reassessment #1: February–April 2011; #2: June–September 2011; #3: October–December 2011; #4: April–June 2012; ref. 33). Recorded beverages and food items were categorized into 50 groups by their nutrient profile or culinary uses, similar to the classification used in the UK National Diet and Nutrition Survey (See Supplementary Table S2). Participants who completed two or more 24-hour dietary assessments were included in the analyses to better approximate usual long-term intakes. The Oxford WebQ automatically generates total nutrient intakes in addition to intakes from each food group or beverage detailed in each assessment. The mean intakes of nutrients and food items were obtained from all available WebQs completed by each participant prior to deriving the DPs by RRR.

#### Assessment of outcome

The primary endpoint for the study was incident colorectal cancer, defined by International Classification of Diseases, Tenth Edition codes. Colorectal cancer ICD-10 codes were grouped to produce the following anatomic subsites: the proximal colon [incorporating the cecum (C18.0), appendix (C18.1), ascending colon (C18.2), hepatic flexure (C18.3), transverse colon (C18.4), and splenic flexure (C18.5)], distal colon [incorporating the descending colon (C18.6) and sigmoid colon (C18.7)], and rectum [incorporating the rectosigmoid junction (C19.0) and rectum (C20.0)]. Malignant colonic neoplasms of unspecified location (C18.9) or overlapping anatomic subsites (C18.8) were only included in overall incident colorectal cancer analyses. Synchronous lesions were coded as the most anatomically proximal cancer. For individuals with multiple colorectal cancer diagnoses, only the earliest case was counted (*n* = 451, prior to exclusions). Incident colorectal cancer cases were identified via linkage with death and hospital registries. The hospital registry–based follow-up ended on January 31, 2021, in England, Scotland, and Wales. Participants contributed to person-years starting from their last dietary assessment until censoring on these dates, the time of colorectal cancer diagnosis, or time of death, whichever came first. The death registry captured all deaths prior to April 30, 2020, in England, Scotland, and Wales.

#### Covariate selection

A directed acyclic graph, drawn *a priori*, helped identify covariates that could obscure an independent relationship between diet and colorectal cancer (Supplementary Fig. S1). These variables were age, sex, smoking status (never, previous, or current), Townsend deprivation index (TDI, in quintiles), total energy intake (kJ, log-transformed), diabetes status (yes or no), family history of colorectal cancer (yes or no), physical activity (low, moderate, or high), obesity status (by World Health Organization body mass index (BMI) cutoffs: underweight, healthy weight, overweight, or obesity), and education (higher degree, any school degree, vocational qualifications, or other). For details on the derivation of these covariates, see Supplementary Table S1.

### Statistical analysis

#### DP derivation

The DPs derived by RRR are described fully elsewhere (24, 25). Participants were assigned a *z*-score for each DP, quantifying the extent to which their dietary intake was representative of each DP relative to others in the cohort. From the output of the RRR model, DP *z*-scores for each respondent were calculated as a linear, weighted combination of all their standardized food group intakes by applying factor loadings distinct to each DP. Increased intake of a food group with a positive factor loading increases a participant’s DP *z*-score, whereas a negative factor loading achieves the opposite. The number of DPs extracted mirrors the number of nutrient-response variables used for the derivation of the DPs. There were four response variables (energy density, saturated fatty acids, fiber density, and free sugars); therefore, four distinct DPs were generated. DPs individually explaining >20% of the variation in the response variables (a prespecified threshold) were retained for analysis. DP1, accounting for 43% of the shared variation, is characterized by high intakes of butter; chocolate and confectionery; low-fiber bread; sugars and preserves; red and processed meats; and alcoholic beverages, as well as low intakes of fruits and vegetables and high-fiber cereals. DP2, accounting for 20% of the shared variation in the response variables, is characterized by high intakes of sugar-sweetened beverages; fruit juices; table sugars and preserves; chocolate and confectionery; and milk-based and powdered drinks as well as low intakes of high-fat cheese, butter, and other animal fat spreads; eggs/egg dishes; red meat; coffee and tea; and processed meats. See Gao and colleagues (25) for the complete list of food items and their respective factor loadings comprising DP1 and DP2 and Supplementary Table S2 for descriptions of the food items. The third and fourth DPs only contributed 10% and 4% of the variation in the response variables, respectively, and thus were not analyzed (25).

#### Associations between DPs and outcomes

Multivariable Cox regression models were built using age as the underlying timescale variable. HRs for incident colorectal cancer were estimated for every one-unit increase in DP *z*-scores as well for *z*-score quintiles (referent: quintile 1) using the floating absolute risk method to stabilize variance in the risk estimates (34).

Proportional hazards assumption violations were assessed by treating each variable’s coefficient as time-varying and addressed by stratification as necessary. Person-time of follow-up was calculated from the age at last dietary assessment completion until the age at which colorectal cancer or censoring occurred. To illustrate how our prespecified covariates abrogate the associations between the DPs and incident colorectal cancer, sequential stratification/adjustments were performed in the following groupings: (i) adjustments for age and sex; (ii) adjustments for behavioral factors (smoking status, physical activity level, and total energy intake) and family history of colorectal cancer; (iii) socioeconomic status (TDI and educational attainment); and (iv) BMI and diabetes status.

Tests for interactions (for sex and age ≤55 vs. >55 years), heterogeneity, and trend were performed using likelihood ratio tests. Restricted cubic splines were generated with five knots to visually examine for nonlinear associations between the DPs and incident colorectal cancer.

#### Sensitivity analyses

To address potential reverse causality of dietary changes due to symptomatic colorectal cancer, cases in the first 3 years of follow-up were excluded. Similarly, we separately excluded participants reporting major dietary changes in the 5 years preceding study entry. Another sensitivity analysis substituted waist circumference for (35) BMI to see if the results were impacted by the metric selected to represent obesity. We repeated the analysis after excluding participants with any history of endoscopic examinations or procedures to mitigate potential outcome misclassification, as a polypectomy (i.e., the surgical or endoscopic removal of colorectal polyps) may have preempted a future colorectal cancer. As participants were only included in this study if they completed at least two or more 24-hour dietary assessments, we assessed the robustness of our findings by restricting the analysis to those who completed at least three 24-hour dietary assessments. The last sensitivity analysis excluded smokers as smoking has been implicated in obesity-related paradoxes in disease associations (35), as well as being a known appetite-suppressant, which could potentially impact diet.

All analyses were performed using Stata MP version 17.0 (StataCorp LP). The UK Biobank study was conducted in accordance with the Declaration of Helsinki, and ethical approval was granted by the North West Multi-Centre Research Ethics Committee (reference number 06/MRE08/65). The UK Biobank was also granted approval from the Research Tissue Bank Ethics Committee (reference number 16/NW/0274). The current research was conducted using the UK Biobank resource under application number 14990. Individuals provided informed written consent to participate and for follow-up through data linkage. No further ethics approval was required for this study.

### Ethics approval and consent to participate

Parent study: The UK Biobank study was conducted in accordance with the Declaration of Helsinki, and ethical approval was granted by the North West Multi-Centre Research Ethics Committee (reference number 06/MRE08/65). The UK Biobank was also granted approval from the Research Tissue Bank Ethics Committee (reference number 16/NW/0274). The current research was conducted using the UK Biobank resource under application number 14990. Individuals provided informed consent to participate and for follow-up through data linkage. No further ethics approval was required for this study.

### Data availability

The datasets generated and/or analyzed for this current study can be made available to researchers who apply to use the UK Biobank datasets. Registration is initiated at http://www.ukbiobank.ac.uk/register-apply.

## Results

### Study sample characteristics

After exclusions, 114,443 participants (55.4% women) ages 40 to 70 years remained for analysis (Supplementary Fig. S2). Over 1,054,488 total person-years and a median of 8.0 years of follow-up, there were 1,089 cases of incident colorectal cancer. Table 1 outlines the colorectal cancer outcomes and baseline demographic, clinical, and dietary characteristics of the participants by quintiles for both DPs.

**Table 1.** Colorectal cancer outcomes, baseline characteristics, and dietary intakes of participants by DP quintiles.

Characteristic	Total*n* = 114,443	DP1, quintiles					DP2, quintiles				
		Quintile 1*n* = 22,816	Quintile 2*n* = 22,948	Quintile 3*n* = 23,024	Quintile 4*n* = 23,077	Quintile 5*n* = 22,578	Quintile 1*n* = 22,712	Quintile 2*n* = 22,953	Quintile 3*n* = 23,023	Quintile 4*n* = 22,931	Quintile 5*n* = 22,824
Total colorectal cancer cases (*n*, %)	1,089 (1.0%)	180 (0.8%)	216 (0.9%)	213 (0.9%)	238 (1.0%)	242 (1.1%)	249 (1.1%)	205 (0.9%)	214 (0.9%)	202 (0.9%)	219 (1.0%)
Colorectal cancer by anatomic location (*n*, %)											
Proximal	396 (0.3%)	71 (0.3%)	86 (0.4%)	76 (0.3%)	92 (0.4%)	71 (0.3%)	89 (0.4%)	66 (0.3%)	82 (0.4%)	77 (0.3%)	82 (0.4%)
Distal	241 (0.2%)	38 (0.2%)	40 (0.2%)	44 (0.2%)	57 (0.2%)	62 (0.3%)	56 (0.2%)	52 (0.2%)	44 (0.2%)	34 (0.1%)	55 (0.2%)
Rectal	380 (0.3%)	61 (0.3%)	67 (0.3%)	79 (0.3%)	77 (0.3%)	96 (0.4%)	95 (0.4%)	71 (0.3%)	74 (0.3%)	72 (0.3%)	68 (0.3%)
Unspecified/overlapping	72 (0.1%)	10 (0.0%)	23 (0.1%)	14 (0.1%)	12 (0.1%)	13 (0.1%)	9 (0.0%)	16 (0.1%)	14 (0.1%)	19 (0.1%)	14 (0.1%)
Sex (*n*, %)											
Female	63,454 (55.4%)	15,855 (69.5%)	14,788 (64.4%)	13,301 (57.8%)	11,552 (50.1%)	7,958 (35.2%)	12,547 (55.2%)	13,897 (60.5%)	13,579 (59.0%)	12,681 (55.3%)	10,750 (47.1%)
Male	50,989 (44.6%)	6,961 (30.5%)	8,160 (35.6%)	9,723 (42.2%)	11,525 (49.9%)	14,620 (64.8%)	10,165 (44.8%)	9,056 (39.5%)	9,444 (41.0%)	10,250 (44.7%)	12,074 (52.9%)
Age, years (SD)	55.9 (7.8)	57.3 (7.3)	56.8 (7.6)	56.2 (7.8)	55.3 (7.9)	53.9 (8.1)	55.9 (7.8)	56.1 (7.7)	56.2 (7.8)	56.1 (7.8)	55.3 (8.1)
BMI, kg/m^2^ (SD)	26.7 (4.6)	26.2 (4.5)	26.3 (4.4)	26.5 (4.4)	26.9 (4.5)	27.6 (4.8)	26.9 (4.9)	26.7 (4.6)	26.7 (4.6)	26.6 (4.4)	26.7 (4.5)
Smoking status (*n*, %)											
Never	66,019 (57.7%)	13,811 (60.5%)	13,669 (59.6%)	13,566 (58.9%)	13,054 (56.6%)	11,919 (52.8%)	12,275 (54.0%)	12,973 (56.5%)	13,400 (58.2%)	13,687 (59.7%)	13,684 (60.0%)
Previous	40,491 (35.4%)	8,130 (35.6%)	8,187 (35.7%)	8,124 (35.3%)	8,313 (36.0%)	7,737 (34.3%)	8,472 (37.3%)	8,443 (36.8%)	8,222 (35.7%)	7,863 (34.3%)	7,491 (32.8%)
Current	7,933 (6.9%)	875 (3.8%)	1,092 (4.8%)	1,334 (5.8%)	1,710 (7.4%)	2,922 (12.9%)	1,965 (8.7%)	1,537 (6.7%)	1,401 (6.1%)	1,381 (6.0%)	1,649 (7.2%)
TDI, quintiles (*n*, %)											
Quintile 1	22,958 (20.1%)	4,672 (20.5%)	4,803 (20.9%)	4,773 (20.7%)	4,639 (20.1%)	4,071 (18.0%)	4,338 (19.1%)	4,741 (20.7%)	4,779 (20.8%)	4,668 (20.4%)	4,432 (19.4%)
Quintile 2	22,958 (20.1%)	4,671 (20.5%)	4,786 (20.9%)	4,630 (20.1%)	4,644 (20.1%)	4,227 (18.7%)	4,362 (19.2%)	4,629 (20.2%)	4,739 (20.6%)	4,700 (20.5%)	4,528 (19.8%)
Quintile 3	22,925 (20.0%)	4,588 (20.1%)	4,553 (19.8%)	4,723 (20.5%)	4,573 (19.8%)	4,488 (19.9%)	4,543 (20.0%)	4,594 (20.0%)	4,660 (20.2%)	4,652 (20.3%)	4,476 (19.6%)
Quintile 4	22,889 (20.0%)	4,487 (19.7%)	4,516 (19.7%)	4,599 (20.0%)	4,623 (20.0%)	4,664 (20.7%)	4,760 (21.0%)	4,575 (19.9%)	4,568 (19.8%)	4,485 (19.6%)	4,501 (19.7%)
Quintile 5	22,713 (19.8%)	4,398 (19.3%)	4,290 (18.7%)	4,299 (18.7%)	4,598 (19.9%)	5,128 (22.7%)	4,709 (20.7%)	4,414 (19.2%)	4,277 (18.6%)	4,426 (19.3%)	4,887 (21.4%)
Educational attainment (*n*, %)											
Higher degree	59,224 (51.7%)	12,946 (56.7%)	12,548 (54.7%)	12,028 (52.2%)	11,608 (50.3%)	10,094 (44.7%)	12,188 (53.7%)	11,950 (52.1%)	11,817 (51.3%)	11,839 (51.6%)	11,430 (50.1%)
Any school degree	33,539 (29.3%)	6,140 (26.9%)	6,584 (28.7%)	6,694 (29.1%)	6,972 (30.2%)	7,149 (31.7%)	6,556 (28.9%)	6,787 (29.6%)	6,870 (29.8%)	6,733 (29.4%)	6,593 (28.9%)
Vocational qualification	14,428 (12.6%)	2,400 (10.5%)	2,433 (10.6%)	2,867 (12.5%)	3,049 (13.2%)	3,679 (16.3%)	2,676 (11.8%)	2,747 (12.0%)	2,802 (12.2%)	2,934 (12.8%)	3,269 (14.3%)
None of the above	7,252 (6.3%)	1,330 (5.8%)	1,383 (6.0%)	1,435 (6.2%)	1,448 (6.3%)	1,656 (7.3%)	1,292 (5.7%)	1,469 (6.4%)	1,534 (6.7%)	1,425 (6.2%)	1,532 (6.7%)
Physical activity group (*n*, %)											
Low	22,280 (19.5%)	3,279 (14.4%)	3,895 (17.0%)	4,504 (19.6%)	5,051 (21.9%)	5,551 (24.6%)	4,572 (20.1%)	4,649 (20.3%)	4,563 (19.8%)	4,307 (18.8%)	4,189 (18.4%)
Moderate	51,546 (45.0%)	9,846 (43.2%)	10,553 (46.0%)	10,564 (45.9%)	10,620 (46.0%)	9,963 (44.1%)	10,405 (45.8%)	10,453 (45.5%)	10,401 (45.2%)	10,312 (45.0%)	9,975 (43.7%)
High	40,617 (35.5%)	9,691 (42.5%)	8,500 (37.0%)	7,956 (34.6%)	7,406 (32.1%)	7,064 (31.3%)	7,735 (34.1%)	7,851 (34.2%)	8,059 (35.0%)	8,312 (36.2%)	8,660 (37.9%)
Diabetes diagnosis (*n*, %)	4,287 (3.7%)	913 (4.0%)	899 (3.9%)	790 (3.4%)	843 (3.7%)	842 (3.7%)	1,192 (5.2%)	989 (4.3%)	852 (3.7%)	693 (3.0%)	561 (2.5%)
Family history of colorectal cancer (*n*, %)	11,528 (10.1%)	2,405 (10.5%)	2,398 (10.4%)	2,317 (10.1%)	2,259 (9.8%)	2,149 (9.5%)	2,388 (10.5%)	2,269 (9.9%)	2,299 (10.0%)	2,329 (10.2%)	2,243 (9.8%)
History of previous endoscopy (*n*, %)	12,028 (10.5%)	2,482 (10.9%)	2,494 (10.9%)	2,368 (10.3%)	2,384 (10.3%)	2,300 (10.2%)	2,253 (9.9%)	2,380 (10.4%)	2,412 (10.5%)	2,434 (10.6%)	2,549 (11.2%)
Energy intake, kJ/day (median, IQR)	8,418.6 (7,180.8–9,819.9)	7,963.0 (6,837.1–9,264.4)	7,939.5 (6,819.9–9,149.3)	8,171.5 (7,034.9–9,436.2)	8,570.2 (7,362.5–9,873.4)	9,648.9 (8,325.5–11,104.0)	8,998.4 (7,732.9–10,455.8)	8,223.4 (7,028.9–9,562.9)	8,087.2 (6,890.4–9,425.9)	8,161.6 (6,962.1–9,487.7)	8,670.7 (7,431.6–10,098.7)
Dietary energy density, kJ/g (SD)	6.5 (1.5)	4.9 (0.8)	5.8 (0.7)	6.4 (0.8)	7.1 (0.9)	8.3 (1.3)	7.05 (1.5)	6.5 (1.4)	6.3 (1.4)	6.3 (1.4)	6.4 (1.6)
Free sugar, %E (SD)	11.4 (4.9)	8.9 (3.9)	10.3 (4.0)	11.3 (4.2)	12.1 (4.6)	14.5 (5.8)	7.8 (3.1)	9.2 (3.3)	10.6 (3.4)	12.6 (3.6)	17.0 (4.9)
Saturated fat, %E (SD)	11.7 (2.9)	9.8 (2.4)	11.1 (2.5)	11.8 (2.6)	12.5 (2.8)	13.3 (3.0)	14.2 (2.7)	12.2 (2.5)	11.3 (2.5)	10.7 (2.5)	10.1 (2.6)
Fiber density, g/MJ (SD)	2.1 (0.6)	2.8 (0.5)	2.3 (0.4)	2.1 (0.4)	1.8 (0.3)	1.5 (0.3)	2.0 (0.5)	2.1 (0.6)	2.2 (0.6)	2.2 (0.6)	2.1 (0.64)

NOTE: Higher degree defined as college, university, or professional degree/qualification. Any school degree defined as A-level, AS-level, O-level, GCSE, or CSE. Vocational qualifications defined as HNC, HND, or NVQ. Physical activity defined using IPAQ or MET scores: low (<600 MET-minutes per week); moderate (≥600 and <3000 MET-minutes per week); high (≥ 3,000 MET-minutes per week); and missing.

Abbreviations: CSE, certificate of secondary education; %E, percent of total energy intake; GCSE, general certificate of secondary education; HNC, higher national certificate, HND, higher national diplomas; IPAQ, International Physical Activity Questionnaire; MET scores, metabolic equivalent scores; NVQ, national vocational qualification.

In higher DP1 quintiles, higher proportions of men, current smokers, lower physical activity, and deprivation as well as higher mean BMI and higher median daily energy intake were observed. Conversely, lower mean age and lower proportions of diabetes, a family history of colorectal cancer, higher education, previous lower endoscopy, and of those reporting major changes to their diets over the last 5 years were observed in higher DP1 quintiles. Increasing proportions of incident overall colorectal, distal colon, and rectal cancers were observed in higher DP1 quintiles. No statistically significant differences in the proportions of proximal colon cancer and unspecified or overlapping colorectal cancer across DP1 quintiles were observed.

In higher DP2 quintiles, higher proportions of never-smokers, high activity levels, and previous endoscopic procedures were observed. Lower proportions of postsecondary education, family history of colorectal cancer, diabetes, and lower mean age were also seen.

Mean DP1 *z*-scores were higher among those who were diagnosed with colorectal cancer compared with noncases [0.142 (SD 1.45) vs. −0.011 (SD 1.45); *P* = 0.001]. No significant differences in DP2 *z*-scores were noted between colorectal cancer cases and noncases (see Supplementary Table S3).

### Association between the DPs and incident colorectal cancer

#### DP1

**Figure 1.**
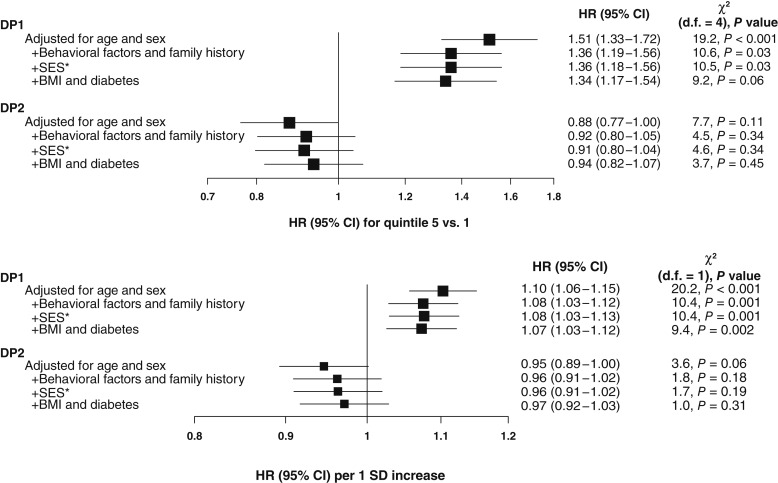
Sequential stratification/adjustments to the model comparing the risk of incident colorectal cancer associated with DP1 and DP2. Top, sequential changes to the HRs for quintiles 5 versus 1 for DP1 and DP2. Bottom, sequential changes to the HRs for DP1 and DP2 *z*-scores in continuous form. The *X*-axis represents HRs on the log-scale. CIs were obtained using the floating absolute risk method (34). χ^2^ and *P* values were calculated by likelihood ratio tests to assess the heterogeneity in the associations with sequential adjustments for covariates. Behavioral factors included smoking status, physical activity level (MET-hours/week), and total energy intake (ln-kJ). SES* comprises TDI and educational attainment. Models were stratified by covariates violating the proportional hazards assumption: physical activity, family history of colorectal cancer, education, and BMI. SES, socioeconomic status.

**Figure 2.**
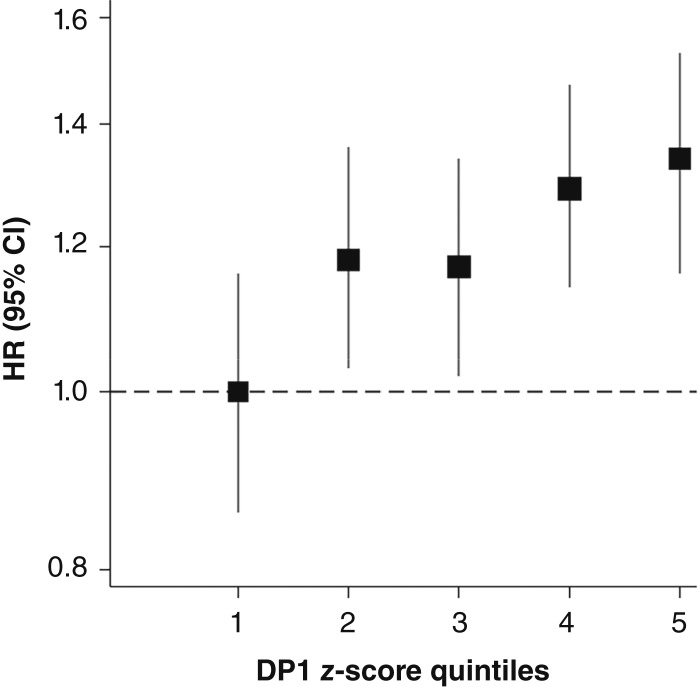
Shape plot of the HRs and 95% CIs denoting incident colorectal cancer risk by DP1 quintiles in the fully adjusted model; DP1 quintile 1 is shown as the reference. CIs were obtained using the floating absolute risk method (34).

**Figure 3.**
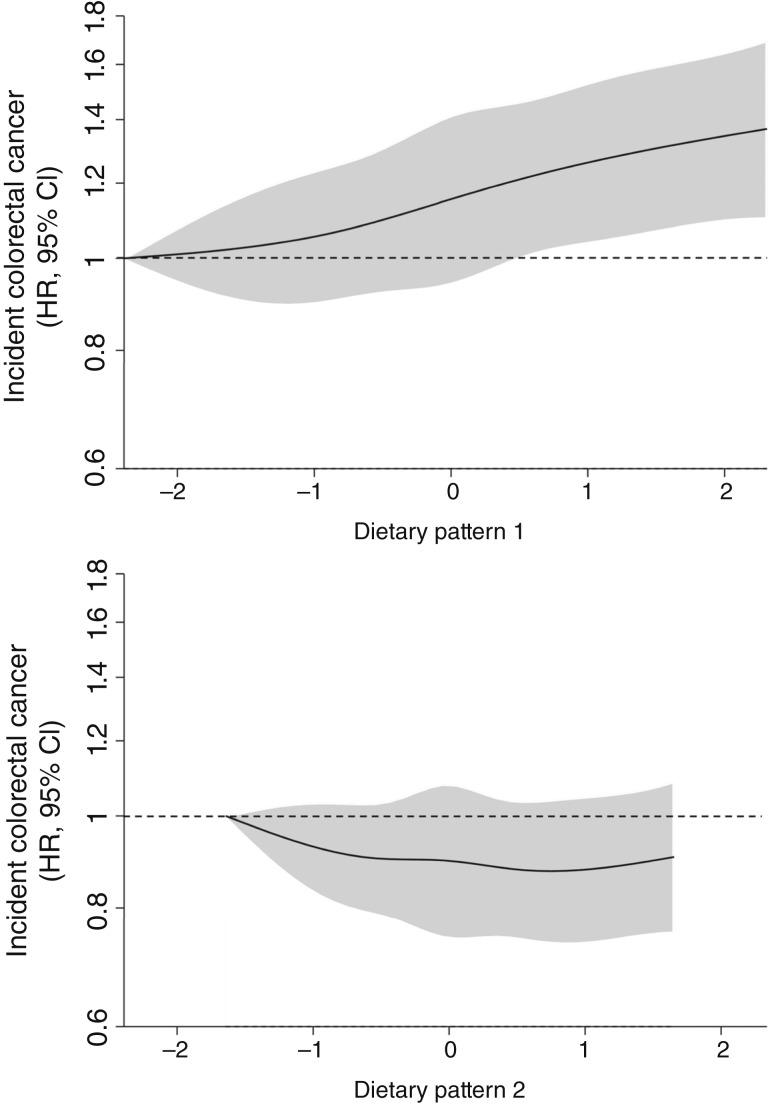
HRs (95% CIs) of continuous form. **A,** DP1 *z*-scores and **B,** DP2 *z*-scores for the risk of incident colorectal cancer. HRs (solid black line) and 95% CIs (gray shading) were derived from spline regression models to evaluate for potential nonlinear associations between DP1 scores and incident colorectal cancer. The reference point of the DP *z*-scores was set to the fifth percentile values. *X*-axis represents DP *z*-scores.

Figure 1 shows the associations between DP1 and incident colorectal cancer with sequential adjustments for covariates. After stratification by family history of colorectal cancer, obesity status, education, and physical activity and adjustment for age, sex, smoking, diabetes, TDI, and total energy, a positive association between DP1 and incident colorectal cancer remained; per one-unit increase in *z*-score (i.e., one SD), participants had a 7% higher risk of incident colorectal cancer [HR_*z*-score_, 1.07; 95% confidence interval (CI), 1.03–1.12]. This association was also positive across DP1 quintiles [χ^2^ = 7.90, degree of freedom (d.f.) = 1, *P*_trend_ = 0.005]. Compared with quintile 1, quintile 5 of DP1 was associated with a 34% increased risk of colorectal cancer (HR, 1.34; 95% CI, 1.17–1.54; Figs. 1 and 2). See Supplementary Table S4 for sequential adjustments to the linear form of DP1 and Supplementary Table S5 for all covariates’ HRs in the fully adjusted model. The splines generated also illustrate a linear association between DP1 and colorectal cancer [Fig. 3 (top)]. Supplementary Table S5 provides the effect estimates of DP1 and the additional covariates.

**Figure 4.**
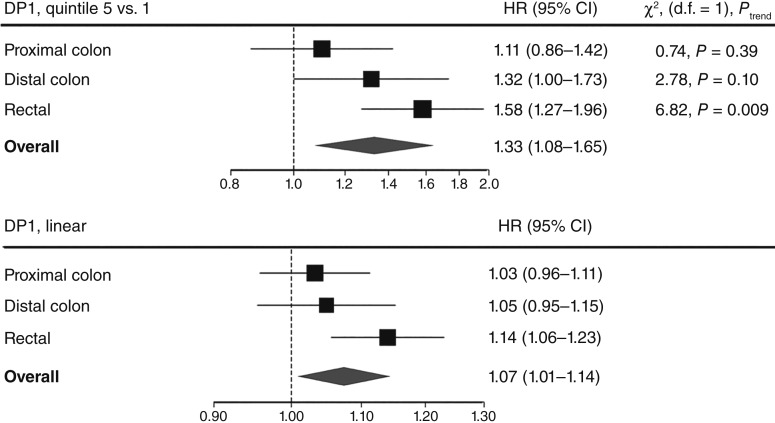
DP1 associations with colorectal cancer by anatomic subsite. Top, HRs of quintile 5 versus quintile 1. Bottom, HRs of DP1 *z*-scores in continuous form. Unspecified (*n* = 68) and overlapping (*n* = 4) colorectal cancers were excluded as they could not be localized to an anatomic subsite. Test for trend down using LRTs. LRT, likelihood ratio test.

Figure 4 illustrates the exploratory tumor subsite analysis for DP1. DP1 was not associated with proximal colon cancer [HR_*z*-score_, 1.03 (95% CI, 0.96–1.11); HR_Q5 vs. Q1_, 1.11 (95% CI, 0.86–1.42); χ^2^ = 0.74, d.f. = 1, *P*_trend_ = 0.3887]. Similarly, there was no linear association between DP1 and incident distal colon cancer (HR_*z*-score_, 1.05; 95% CI, 0.96–1.15) and no trend across quintiles (χ^2^ = 2.78, d.f. = 1, *P*_trend_ = 0.095), although quintile 5 versus 1 was borderline significant (HR_Q5 vs. Q1_, 1.32; 95% CI, 1.00–1.73). In contrast, compared with incident overall colorectal, proximal, and distal colon cancers, a stronger association was observed for rectal cancer [HR_*z*-score_, 1.14 (95% CI, 1.06–1.23); HR_Q5 vs. Q1_, 1.58 (95% CI, 1.27–1.96); χ^2^ = 6.82, d.f. = 1, *P*_trend_ = 0.009]. Supplementary Table S6 provides the HRs and 95% CIs for all DP1 quintiles by anatomic subsite.

There were no interactions between DP1 with age ≤55 years versus >55 years or sex (Supplementary Fig. S3). Table 2 demonstrates the effect estimates of DP1 and DP2 for incident colorectal cancer for both the male and female subgroups.

**Table 2.** Associations of DP1 and DP2 with incident colorectal cancer by sex subgroups.

	DP1			DP2		
	All colorectal cancer cases	Women only	Men only	All colorectal cancer cases	Women only	Men only
Total participants, *n*	114,443	63,454	50,988	114,443	63,454	50,988
Cases, *n*	1,089	462	627	1,089	462	627
DP *z*-scores, linear form	1.07 (1.03–1.12)	1.05 (0.98–1.13)	1.08 (1.02–1.15)	0.97 (0.92–1.03)	0.99 (0.90–1.08)	0.96 (0.90–1.03)
DP *z*-score quintiles						
Quintile 1	Ref.	Ref.	Ref.	Ref.	Ref.	Ref.
Quintile 2	1.18 (0.97–1.44)	1.38 (1.06–1.79)	0.99 (0.73–1.34)	0.86 (0.71–1.04)	0.81 (0.61–1.07)	0.92 (0.71–1.18)
Quintile 3	1.17 (0.96–1.43)	1.15 (0.86–1.52)	1.17 (0.88–1.55)	0.90 (0.75–1.08)	0.96 (0.73–1.26)	0.84 (0.66–1.09)
Quintile 4	1.29 (1.06–1.57)	1.43 (1.08–1.91)	1.18 (0.90–1.56)	0.85 (0.71–1.03)	0.72 (0.53–0.97)	0.96 (0.76–1.23)
Quintile 5	1.34 (1.09–1.64)	1.19 (0.83–1.69)	1.32 (1.03–1.74)	0.94 (0.78–1.12)	1.05 (0.79–1.40)	0.87 (0.68–1.10)
LRT χ^2^ for DP *z*-scores, linear form^a^	χ^2^ = 9.43, d.f. = 1, *P* = 0.0026	χ^2^ = 1.86, d.f. = 1, *P* = 0.1732	χ^2^ = 7.22, d.f. = 1, *P* = 0.0072	χ^2^ = 1.02, d.f. = 1, *P* = 0.3128	χ^2^ = 0.09, d.f. = 1, *P* = 0.7621	χ^2^ = 1.09, d.f. = 1, *P* = 0.2970
Test for trend across DP *z*-score quintiles^a^	χ^2^ = 7.90, d.f. = 1, *P* = 0.005	χ^2^ = 2.02, d.f. = 1, *P* = 0.1551	χ^2^ = 5.90, d.f. = 1, *P* = 0.0151	χ^2^ = 0.52, d.f. = 1, *P* = 0.4698	χ^2^ = 0.01, d.f. = 1, *P* = 0.9145	χ^2^ = 0.79, d.f. = 1, *P* = 0.3741
DP *z*-score quintiles, floating absolute risk method						
Quintile 1	1.00 (0.86–1.16)	1.00 (0.82–1.22)	1.00 (0.80–1.25)	1.00 (0.88–1.14)	1.00 (0.82–1.21)	1.00 (0.85–1.18)
Quintile 2	1.18 (1.03–1.36**)**	1.38 (1.16–1.65)	0.99 (0.80–1.22)	0.86 (0.75–0.99)	0.81 (0.66–0.99)	0.92 (0.76–1.10)
Quintile 3	1.17 (1.02–1.34)	1.15 (0.93–1.41)	1.17 (0.98–1.39)	0.90 (0.78–1.03)	0.96 (0.79–1.16)	0.84 (0.70–1.02)
Quintile 4	1.29 (1.14–1.47)	1.43 (1.17–1.76)	1.18 (1.01–1.39)	0.85 (0.74–0.98)	0.72 (0.57–0.91)	0.96 (0.81–1.15)
Quintile 5	1.34 (1.16–1.53)	1.19 (0.89–1.59)	1.32 (1.13–1.54)	0.94 (0.81–1.07)	1.05 (0.85–1.30)	0.87 (0.73–1.03)

NOTE: Adjusted HRs and 95% CIs of total DP *z*-scores (linear form) were obtained using Cox proportional hazard regression. Adjusted HRs and 95% CIs of DP *z*-score quintiles were obtained using Cox proportional hazard regression (upper half of the table). CIs obtained using the floating absolute risk method are presented in the bottom half of the table. The model used was adjusted for age at baseline (not attained age at diagnosis or censoring), smoking status, total daily energy intake (log-kJ), TDI (quintiles), and diabetes status. The model was also stratified by BMI (underweight, healthy weight, overweight, or obese), physical activity level (MET-hours per week: low, moderate, or vigorous), educational attainment (higher degree, any school degree, vocational qualification, or none of the above), and family history of colorectal cancer.

The sex-specific effect estimates for DP1 and DP2 listed (calculated from male and female subgroups) will differ slightly from those in Supplementary Fig. S3, which are obtained by testing for interaction between sex and the DPs in the original models.

Abbreviation: LRT, likelihood ratio test.

a χ^2^ values were calculated by LRT, to measure the extent to which the DP is associated with incident overall colorectal cancer in the sequentially adjusted and stratified models (i.e., comparing each model with and without the DP).

#### DP2

In the fully adjusted model, there was no linear positive association between DP2 and incident colorectal cancer [HR_*z*-score_, 0.97 (95% CI, 0.92–1.03); HR_Q5 vs. Q1_, 0.94 (95% CI, 0.81–1.07); trend: χ^2^ = 0.52, d.f. = 1, *P*_trend_ = 0.47]. See Supplementary Tables S7 for the sequential adjustments for the DP2 model and Supplementary Table S8 for all covariate HRs in the final model examining DP2. The splines generated for DP2 further illustrates a nonsignificant U-shaped association with incident colorectal cancer [Fig. 3 (bottom)]. There were no interactions between DP2 and age or sex (Supplementary Fig. S3), and there were no significant associations for DP2 with colorectal cancer by anatomic subgroup (Supplementary Fig. S4).

### Sensitivity analyses

#### DP1

**Figure 5.**
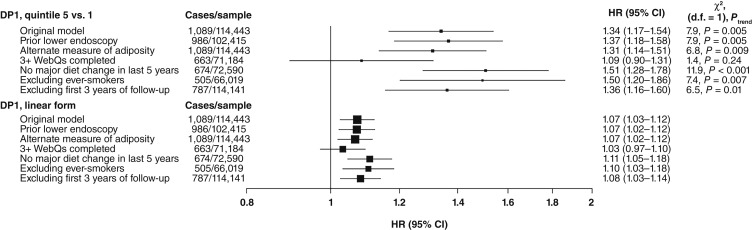
DP1 sensitivity analyses. The *x*-axis comprises HRs and associated 95% CIs and is on the log-scale. CIs presented were obtained using the floating absolute risk method. WebQ, Oxford WebQ 24-hour dietary assessment tool.

In our sensitivity analyses for DP1, the magnitude of the HRs did not materially change except for the analysis restricted to participants who completed three or more WebQs, in which the overall linear association was no longer observed (HR_*z*-score_, 1.03; 95% CI, 0.97–1.10; see Supplementary Table S9 for additional results of the sensitivity analyses; see Fig. 5).

#### DP2

In the sensitivity analyses conducted for DP2, no significant linear associations with incident colorectal cancer emerged (Supplementary Fig. S5 and Supplementary Table S10).

## Discussion

DP1, previously identified from a large middle-aged British cohort and found to have significant associations with incident T2DM, CVD, and all-cause mortality, was found to be significantly associated with incident colorectal cancer. This DP, characterized by high intakes of chocolate and confectionery; butter; low-fiber bread; added sugars; and red and processed meats and low intakes of fruits, vegetables, and high-fiber cereals, was positively associated with incident colorectal cancer, with a 34% increased risk in the highest quintile when compared with the lowest quintile. Interestingly, the association between DP1 and incident colorectal cancer persisted after accounting for BMI (obesity) and diabetes, implying that the effects of DP1 on colorectal cancer risk are not completely explained by DP1’s previously identified associations with these metabolic diseases (25).

The subgroup analysis by tumor subsite revealed no linear association for proximal or distal colon cancers, whereas higher DP1 *z*-scores were strongly associated with rectal cancer. However, a test of homogeneity did not confirm statistically significant differences in risk estimates between tumor subsites.

We did not identify any interactions with sex or age, suggesting this pattern may be relevant for the whole population (6, 36). However, the lack of interaction with age might be partially explained by our study sample’s demographics and a lack of power for this specific question; only 68 participants were ≤55 years of age at the time of colorectal cancer diagnosis. Our study thus highlights the need for future studies on diet and colorectal cancer associations to be conducted on younger populations.

The results from the sensitivity analyses supported the findings of our main model, except for the analysis restricted to participants who completed 3+ WebQs, in which no linear association was observed between DP1 and incident colorectal cancer. Although this discrepancy might be partially explained by differences in the recorded baseline characteristics of participants who completed 3+ versus 2 WebQs, any observed differences are nearly negligible (Supplementary Table S11). Rather, the observed loss of association may instead be an issue of smaller sample size (and therefore loss of power), as restricting the analysis to those who completed only two WebQs represents a 38% reduction in sample size (43,259/114,443) and a 39% reduction in colorectal cancer cases (426/1,089).

In contrast to DP1, we did not identify a significant linear association between incident colorectal cancer and DP2, which was characterized by high intakes of sugar-sweetened beverages; fruit juices; table sugars and preserves; chocolate and confectionery; and milk-based and powdered drinks as well as low intakes of high-fat cheese, butter, and other animal fat spreads; eggs/egg dishes; red meat; coffee and tea; and processed meats. Our results here mimic the lack of linear associations between DP2 and CVD and incident T2DM from previous studies (23, 25). The lack of association between DP2 and colorectal cancer may be explained by the fact that DP2 represents a more moderate diet. In other words, it could be because the magnitudes of the factor loadings for food items thought to reduce colorectal cancer risk may be opposing the factor loadings of food items thought to increase colorectal cancer risk in a way that abrogates an overall association between DP2 and incident colorectal cancer.

Although an underlying biological pathway linking diet to colorectal cancer remains uncertain, multiple mechanisms have been proposed. Some food items have been more thoroughly examined than others. For instance, the World Health Organization classifies processed and red meats as definite and probable carcinogens, respectively. The mechanisms underlying the carcinogenicity of red and processed meats have been well studied and include: (i) the mutagenic effects of heterocyclic amines and polycyclic aromatic hydrocarbons generated while cooking these meats; (ii) heme- and heme-iron–mediated proliferation of DNA-damaging agents (e.g., reactive oxygen species, lipid peroxidation degradation products such as reactive aldehydes, and heme-dependent generation of N-nitroso compounds); and (iii) heme-iron–induced gut dysbiosis (37).

Although DP1 is characterized by positive factor loadings for red and processed meats, the strongest positive factor loadings were from multiple food items that would fall under the description of “ultraprocessed foods” (UPF), which are typically energy-dense, high in sugar, salt, and/or fat, and low in dietary fiber. Examples of DP1 food items that are considered to be UPFs include chocolate and confectionery; sugar-sweetened beverages; crisps and savory snacks; grain-based desserts; and table sugars and preserves (processed meats are also considered to be UPFs). Increased intakes of these items define the DP1 profile, with chocolate and confectionery constituting the strongest positive factor loading for DP1. UPFs are also common constituents of the so-called “Western” diets. In a recent prospective cohort study combining the Health Professionals Follow-up Study and Nurses’ Health Study, men in the highest quintile of UPF consumption were found to have a 29% increased risk of incident colorectal cancer compared with the lowest quintile (no association was found for women; ref. 38).

One suggested pathway links UPFs in Western diets to gut dysbiosis and inflammation, leading to altered gut barrier function and facilitating oncogene expression (39). As an example, dysbiosis-induced changes to short-chain fatty acid (SCFA) production via intestinal bacterial fermentation of substrates, including insoluble dietary fiber, have been suggested as one such a mechanism. As SCFAs may have antitumorigenic effects (40), decreased luminal SCFA concentrations may facilitate colorectal neoplasia. Other potential mechanisms include proinflammatory effects of artificial sweeteners and emulsifiers such as carboxymethylcellulose found in UPFs (41, 42) as well as the direct carcinogenic effects of additives or compounds generated during food processing (such as sodium nitrite, which can promote N-nitroso compound formation). Free sugars, a component of UPFs, may contribute to carcinogenesis by the production of certain advanced glycation end products (proteins or lipids that undergo nonenzymatic glycation when exposed to reducing sugars; ref. 43). Sustained inflammation, which is known to be carcinogenic, is promoted by certain advanced glycation end products (43, 44).

Our finding of the association of DP1 with incident overall colorectal cancer is generally supported by other studies examining DPs with overlapping features of Western diets (while noting DPs from other studies were mostly derived using other methodologies). These findings are well-summarized in recent reviews (6, 14). Although some studies found no association between DPs and overall incident colorectal cancer (17, 45–48), several such studies had small case numbers (range: 172–460; refs. 17, 47, 48).

Our analysis of colorectal cancer subsites found no associations of DP1 with proximal or distal colon cancers while observing a stronger association with rectal cancer. This is the inverse of the summary findings from Garcia-Larsen and colleagues (14) meta-analysis of “Western” DPs derived by principal component analysis, who observed associations with colon but not rectal cancer. However, it should be noted that there was considerable heterogeneity across the studies included in their meta-analysis for rectal cancer (*I*^2^ = 62.8%), and our results find support in other studies (7, 49, 50). In one such cohort study of 137,217 American health professionals, individuals in the highest quartile of a “Western” diet—derived by principal component analysis and characterized by red and processed meats, high-fat dairy products, desserts, and refined grains—had a 31% higher risk of incident colorectal cancer compared with those in the lowest quartile (7). Furthermore, their subgroup analysis by tumor location revealed an association between the Western diet and distal colon and rectal cancers but not with proximal colon cancers. Despite using a different derivation method, the DP from this study had reasonable overlap with ours, revealing a similar magnitude of association for overall incident colorectal cancer.

This study has several additional strengths. A key advantage of RRR-derived DPs is that, as a hybrid method, RRR combines *a priori* knowledge of nutrients or biomarkers associated with a given disease with data-driven approaches to produce food-based DPs. This permits a “whole-diet” approach, reducing confounding common to single-nutrient or food studies while simultaneously accounting for potential mechanistic aspects of the response variables. These DPs can provide policymakers with evidence based on food items as opposed to nutrients, in turn facilitating food-based recommendations (24).

Additional strengths of this study include prospective data collection for many variables (e.g., BMI, waist circumference, and smoking status), thus partially addressing recall error and bias. Additionally, although potential selection bias of health-conscious volunteers should be acknowledged, excellent data linkage with UK hospital records in a predominantly single-payer healthcare system implies reasonable representation of the population outcomes by our cohort. Furthermore, as discussed above, the dietary assessment tool used for this study (WebQ) has been validated against biomarkers and exhibits good reproducibility when at least two assessments are performed (32). Additionally, we controlled for important confounders, demonstrating that the DP was not simply a mediator of socioeconomic status and that the DP remained positively associated with colorectal cancer after adjusting for obesity and diabetes, both of which are independent colorectal cancer risk factors (9). Reverse causality was limited by commencing follow-up once the dietary assessments were completed, and a sensitivity analysis excluding participants reporting major dietary changes (perhaps due to colorectal cancer symptoms) and the 3-year lagged analysis after the last dietary assessment support the robustness of our findings.

Despite its strengths, this study has limitations. Although the investigation of RRR-derived DPs facilitates practical, “whole-diet” advice, it is not intended to isolate the effects of specific food items. As with any questionnaire or interview, the WebQ is vulnerable to recall errors, and social desirability bias may have influenced participant’s dietary habits. Furthermore, the WebQ is reliant on the accuracy of the food composition database used to derive the nutrient composition of each food item. As well, uncommon food items were not captured in the WebQ and might contribute to residual confounding. Additionally, although repeated administrations of the WebQ were employed to approximate “usual” long-term intakes, it may not capture a lifetime of dietary habits and exposure. This was partially addressed by our sensitivity analysis of major dietary changes in the 5 years preceding entry into the UK Biobank cohort. Although the association of DP1 with incident colorectal cancer remained unchanged for those who reported no major diet change (cases = 674, *n* = 71,843), the association was no longer present in those reporting a major dietary change (cases = 415, *n* = 41,438). Cautious interpretations of these findings are that a relatively short exposure period to DP1 may not be associated with incident colorectal cancer and that the findings of our main model may indeed reflect long-term dietary information as far back as 5 years before participant entry into the study. Our study sample was 90.0% Caucasian, thus limiting the generalizability to other ethnic groups. Additionally, family history of colorectal cancer from the UK Biobank was limited to first-degree relatives only.

In summary, among UK Biobank participants ages 40 to 70 years, higher *z*-scores for DP1—characterized by high intakes of chocolate and confectionery; butter; low-fiber bread; added sugars; and red and processed meats and low intakes of fruits, vegetables, and high-fiber cereals—are associated with higher risk of incident colorectal cancer, with a stronger association observed for rectal cancers. Reducing population adherence to diets reflecting DP1 may reduce the risk of colorectal cancer in addition to T2DM and CVD, as identified in prior studies.

## Supplementary Material

Supplementary Table S1 Table S1 Derivation of outcome, exposure of interest, model covariates, exclusion criteria, and sensitivity analysis variables from U.K. Biobank data

Supplementary Table S2 Table S2 Food groupings and their constituent food items

Supplementary Table S3 Table S3. Univariable associations with colorectal cancer

Supplementary Table S4 Table S4 Sequential adjustment of the HRs with 95% CIs for DP1’s association with incident CRC.

Supplementary Table S5 Table S5 Hazard ratios and 95% confidence intervals of all variables in the fully-adjusted model for DP1

Supplementary Table S6 Table S6 Subgroup analysis of the association between DP1 and incident CRC by anatomic subgroup, fully adjusted and stratified model.

Supplementary Table S7 Table S7 Sequential adjustment of the HRs with 95% CIs for DP2’s association with incident CRC

Supplementary Table S8 Table S8 Hazard ratios and 95% confidence intervals of all variables in the fully-adjusted model for DP2

Supplementary Table S9 Table S9 Sensitivity analyses of the fully adjusted and stratified model for the association between dietary pattern 1 and incident overall CRC

Supplementary Table S10 Table S10 Sensitivity analyses of the fully adjusted model for the association between dietary pattern 2 and incident overall CRC

Supplementary Table S11 Table S11. Comparison of incident colorectal cancer cases and baseline characteristics between those completing 2 vs. 3+ WebQs

Supplementary Figure S1 Figure S1 Directed acyclic graph of variables used in the model.

Supplementary Figure S2 Figure S2 U.K. Biobank participant exclusion flow chart

Supplementary Figure S3 Figure S3 Hazard ratios and 95% confidence intervals for interactions of DP1 and DP2 with age and sex

Supplementary Figure S4 Figure S4 DP2 associations with CRC by anatomic subsite

Supplementary Figure S5 Figure S5 DP2 Sensitivity analyses
