# Supplementary material for: Associations between Dietary Patterns and Incident Colorectal Cancer in 114,443 Individuals from the UK Biobank: A Prospective Cohort Study
Source: Cancer Epidemiol Biomarkers Prev. 2024 Aug 19;33(11):1445–55. doi: 10.1158/1055-9965.EPI-24-0048 (PMC11528196; doi:10.1158/1055-9965.EPI-24-0048)
Supplement: Supplementary Table S1 — Table S1 Derivation of outcome, exposure of interest, model covariates, exclusion criteria, and sensitivity analysis variables from U.K. Biobank data [file epi-24-0048_supplementary_table_s1_suppst1.docx]

***Table S1:*** *Derivation of outcome, exposure of interest, model covariates, exclusion criteria, and sensitivity analysis variables from U.K. Biobank data.*

| **Variables and rationale** | **Categories or format of values used in analysis** | **UK Biobank variable used (field ID/appropriate code and source)** |
| --- | --- | --- |
| ***Health outcome*** |  |  |
| Colorectal cancer (CRC) | No; Yes | ICD-10 codes:   - *Proximal colon:* C18.0 (caecum), C18.1 (appendix), C18.2 (ascending colon), C18.3 (hepatic flexure), C18.4 (transverse colon), C18.5 (splenic flexure) - *Distal colon:* C18.6 (descending colon), C18.7 (sigmoid colon) - *Rectum:* C19.0 (rectosigmoid junction), C20.0 (rectum) - *Non-localizable tumours:* C18.8 (overlapping anatomic subsites), C18.9 (unspecified location)   Sources:   - HES dataset (IDs: s_diag_icd10_diag1-21) - UKBB dataset (ID: 40001, 40002) |
| Date of CRC diagnosis | Date of diagnosis or death | Sources:   - HES dataset (ID: “admidate” used preferentially; if not available, “epistart” used instead - UKBB dataset (ID: 40000) |
| ***Exposure and nutrition-related variables*** | | |
| Dietary pattern | 1. Quintiles (Q1: lowest quintile of z-scores; Q5: highest) 2. z-score value (for linear analysis) | 1. Source: UKBB dataset (ID: scorex1_5, scorex2_5) 2. Source: UKBB dataset (ID: z_score_factor1, z_score_factor2 |
| Number of Oxford WebQs completed | 2 (minimum required for eligibility); 3; 4; 5. | Source: UKBB dataset (ID: 105010) |
| Total daily energy intake, kJ, log-transformed | Log-kJ | Source: UKBB dataset, log-transformation of total energy intake (ID: 100002) |
| ***Demographics*** | | |
| Age | Years | Age at recruitment  Sources: UKBB dataset (ID: 21022^a^) |
| Sex | Female ; Male. | Source: UKBB dataset (ID: 31^a^) |
| ***Socioeconomic status*** |  |  |
| Townsend deprivation index (TDI) | Quintiles (Q1, lowest quintile of deprivation; Q5, highest quintile of deprivation) | Source: UKBB dataset (ID: 189^a^)  TDI combines levels of: unemployment, non-car ownership, non-home ownership, and household overcrowding as a means of measuring neighbourhood material deprivation and has seen widespread use.  The unemployment and overcrowding percentages (+1) are then log-transformed to normalise the raw values, which tend to be highly skewed. All four variables are subsequently standardized using a Z-score. These four standardized scores are then summed to obtain a single value which is the TDI. Positive values of the index will indicate areas with high material deprivation, whereas those with negative values will indicate relative affluence. A score of 0 represents an area with overall mean values^2^. |
| Educational attainment | Higher degree (college, university, or professional degree/qualification); Any school degree (A-level, AS-level, O-level, GCSE, CSE); Vocational qualifications (National Vocational Qualification, Higher National Diplomas/Certificates); None of the above; Missing | Source: UKBB dataset (ID: 6138^b^) |
| ***Behavioural risk factors*** | | |
| Smoking status | Never; Previous; Current; Missing | Source: UKBB dataset (ID: 20116^b^) |
| Physical activity (International Physical Activity Questionnaire MET scores) | Low (<600 metabolic equivalent (MET)-minutes per week); Moderate (≥600 and <3000 MET-minutes per week); High (≥ 3000 MET-minutes per week); Missing | Source: UKBB dataset (Number of days/week of vigorous physical activity 10+ minutes (ID: 904)^b^; Duration of vigorous activity (ID: 914)^b^; Number of days/week of moderate physical activity 10+ minutes (ID: 884)^b^; Duration of moderate activity (ID: 894)^b^; Number of days/week walked 10+ minutes (ID: 864)^b^; Duration of walks (ID: 874)^b^ |
| ***Health/family history and medical conditions*** |  |  |
| BMI, World Health Organization (WHO) categories | <18.5; 18.5 – 24.9; 25 – 29.9; 30 – 34.9; 35 – 39.9; 40+ | Source: UKBB dataset (ID: 21001^c^) |
| Family history of colorectal cancer | No; Yes | Source: UKBB dataset (ID: 20107^b^, illnesses of father; 20110^b^, illnesses of mother; 20111^b^, illnesses of siblings); thus first degree relatives only |
| Diabetes status (excluding gestational/pregnancy-related diabetes) | No; Yes; Missing | Sources:   1. UKBB dataset (ID: 2443^b^, diabetes diagnosed by doctor; 6153^b^, use of diabetes medications; 2. HES dataset (ID: “s_diag_icd10_diag”; ICD-10 codes: O24.0, O24.1, O24.2, O24.3, E10, E11, E12, E13, E14 |
| ***Exclusions*** | | |
| Personal history of cancer (excluding non-melanoma skin cancer) | No; Yes | Source: UKBB dataset (ID: 20001^e^) |
| Personal history of ulcerative colitis | No; Yes | ICD-10 codes:   - K51.0, K51.1, K51.2, K51.3, K51.4, K51.5, K51.6, K51.8, K51.9   Sources:   1. HES dataset (ID: s_diag_icd10_diag for diagnosis; ) 2. UKBB dataset (ID: 20002^d^ for diagnosis; 20008^#^ for date of diagnosis) |
| History of proctocolectomy | No; Yes | OPCS-4 codes:   - H04.1 , H04.2, H04.3, H04,8 H04.9   Source: HES dataset (ID: “s_oper4_sec” for procedure codes; “opdate_sec” for date of procedure; “admidate” or “epistart” for date of diagnosis) |
| Implausible caloric intakes | For women: < 600 or > 3,500 kcal/day  For men: < 800 or > 4,000 kcal/day | Source: UKBB dataset (the mean total energy intake obtained from instances of ID: 100002). Variable energymean was first converted from kilojoules to kcal prior to applying the exclusion. |
| ***Sensitivity analyses*** | | |
| Prior lower gastrointestinal tract endoscopy | No; Yes | OPCS-4 codes:   - H20.7, H20.8, H20.9, H22.1, H22.8, H22.9, H23.1, H23.2, H23.3, H23.4, H23.5, H23.6, H23.7, H23.8, H23.9, H25.1, H25.2, H25.8, H25.9, H26.1, H26.2, H26.3, H26.4, H26.5, H26.6, H26.7, H26.8, H26.9, H28.1, H28.8, H28.9   Source: HES dataset (ID: “s_oper4_sec” for procedure codes; “opdate_sec” for date of procedure; “admidate” or “epistart” for date of diagnosis) |
| 3+ WebQs completed | Integer | Source: UKBB dataset (ID: 20077**^d^**) |
| Excluding smokers | Selected ‘Never’ smokers (see above) | Source: UKBB dataset (ID: 20116^b^) |
| Self-reported major dietary change in last 5 years | No; Yes (summary value representing both “Yes, due to illness” and “Yes, due to other reasons”) ; Missing | Source: UKBB dataset (ID: 1538^b^) |
| Alternate measure of adiposity: waist circumference | Numerical value | Source: UKBB dataset (ID: 48^c^); waist circumference was measured in a standardized manner by trained assessors. |
| Note: ^a^Recruitment questions, ^b^Touchscreen questions, ^c^Physical measurements, **^d^**24-h online dietary assessment questionnaire, ^e^Verbal interview. Hospital episodes statistics (HES) data was provided in a bespoke dataset file named “HES_raw data.dta”. U.K. Biobank data was provided in a bespoke dataset file named “ DP_UKBB data.dta”. Abbreviations: CSE, certificate of secondary education; GCSE, general certificate of secondary education; HES, Hospital Episode Statistics; UKBB, U.K. Biobank; ICD-10: International Classification of Disease, 10^th^ Edition; BMI, body mass index; OPCS-4, Office of Population Census and Surveys Classification of Interventions and Procedures, Version 4; MET, Metabolic Equivalent. | | |
