# Supplementary material for: Associations between Dietary Patterns and Incident Colorectal Cancer in 114,443 Individuals from the UK Biobank: A Prospective Cohort Study
Source: Cancer Epidemiol Biomarkers Prev. 2024 Aug 19;33(11):1445–55. doi: 10.1158/1055-9965.EPI-24-0048 (PMC11528196; doi:10.1158/1055-9965.EPI-24-0048)
Supplement: Supplementary Table S2 — Table S2 Food groupings and their constituent food items [file epi-24-0048_supplementary_table_s2_suppst2.docx]

***Table S2:*** *Food groupings and their constituent food items.*

| Food groupings | Food items |
| --- | --- |
| High-fat milk and cream | Whole milk >3.6 g fat per 100 g |
|  | Cream |
|  | Full fat yogurt |
| Low-fat milk | Semi skimmed milk >1 g fat per 100 g |
|  | Skimmed milk |
|  | Low fat yogurt |
| Non-dairy milk | Rice/oat milk |
|  | Soya |
| High fat cheese | High fat cheese |
| Low fat cheese | Low fat cheese |
| Olive oil | Olive oil (drizzling/dunking) |
| Poultry | Poultry |
| Red meat | Pork |
|  | Beef |
|  | Lamb |
|  | Other meat |
| Processed meat | Processed meat |
| Oily fish | Oily fish |
| Other fish | Shellfish |
|  | White fish & tinned tuna |
| Coated or breaded meat and fish | Breaded/battered chicken |
|  | Breaded/battered fish |
| Egg & egg dishes | Egg & egg dishes |
| Meat substitutes | Meat substitutes - vegetarian |
|  | Meat substitutes - soy |
| Legumes & pulses | Legumes & pulses |
| Vegetables | Raw salad |
|  | Green leafy/cabbages |
|  | Root vegetables |
|  | Tomatoes |
|  | Allium vegetables |
|  | Other vegetables, including mushrooms, fruiting and mixed vegetables |
|  | Peas/sweetcorn |
| Boiled or baked potatoes | Potatoes/Sweet potatoes (baked/boiled) |
|  | Mashed potatoes |
| Fried or roast potatoes | Fried/roast potatoes |
| Low-fibre bread | White bread |
| High-fibre bread | Wholemeal bread |
|  | Mixed (50/50), brown & seeded |
| Other bread products | Other bread |
|  | Savoury crackers |
|  | Grain dishes - added fat |
|  | Samosa, pakora |
| Fresh fruit | Citrus |
|  | Berries |
|  | Apples & pears |
|  | Other fruit |
| Dried and stewed fruit | Dried fruit |
|  | Stewed fruit |
| Pasta and rice | White pasta & rice |
|  | Sushi |
| Wholemeal pasta, brown rice & other wholegrains | Wholemeal pasta, brown rice & other wholegrains |
| Pizza | Pizza |
| Nuts & seeds | Salted nuts & seeds |
|  | Unsalted nuts & seeds |
| Fruit juice | Fruit juice |
| Coffee and tea | Coffee, caffeinated |
|  | Coffee, decaffeinated |
|  | Tea |
|  | Tea, decaffeinated |
| Sugar-sweetened beverages (SSBs) & other sugary drinks | Sugar-sweetened beverages & other sugary drinks |
| Low/non sugar SSBs | Low/non sugar SSBs |
| Alcoholic drinks (wine, beer, spirits) | White wine |
|  | Red wine |
|  | Fortified wine |
|  | Beer & Cider |
|  | Spirits |
| Water/Sparkling water | Water/Sparkling water |
| Milk-based and powdered drinks | Milk-based and powdered drinks |
| Soups | Soups |
| Sauces & condiments (high fat) | Sauces & condiments (high fat) |
| Sauces & condiments (low fat) | Sauces & condiments (low fat) |
| Milk-based desserts | Milk-dairy desserts |
|  | Soy desserts and yogurt |
| Grain-based desserts | Other desserts & cakes & pastries |
| Table sugars & preserves | Table sugars & preserves |
| Chocolate and confectionery | Chocolate confectionery |
|  | Other sweets |
| High-fibre breakfast cereals | Bran cereal |
|  | Biscuit cereal |
|  | Porridge |
| Other breakfast cereals | Oat cereal (sugar) |
|  | Muesli |
|  | Other cereal (sugar) |
| Crisps and savoury snacks | Savoury snacks (e.g. potato crisps) |
| Vegetable side dishes and dips | Vegetable side dishes |
|  | Vegetable dips |
| Low animal fat spread | Reduced fat animal fat spread |
| Butter and other animal fat spreads | Butter and other animal fat spreads |
| Lower plant-based fat spread | Reduced fat plant-based fat spread |
| Normal plant-based fat spread | Plant-based fat spread (e.g. margarine) |
| Nut-based spread | Nut-based spread (e.g. peanut butter) |
