# Supplementary material for: Associations between Dietary Patterns and Incident Colorectal Cancer in 114,443 Individuals from the UK Biobank: A Prospective Cohort Study
Source: Cancer Epidemiol Biomarkers Prev. 2024 Aug 19;33(11):1445–55. doi: 10.1158/1055-9965.EPI-24-0048 (PMC11528196; doi:10.1158/1055-9965.EPI-24-0048)
Supplement: Supplementary Table S3 — Table S3. Univariable associations with colorectal cancer [file epi-24-0048_supplementary_table_s3_suppst3.docx]

**Table S3.** *Univariable associations with colorectal cancer*

|  | **Non-CRC cases** | **CRC cases** | **Total** | **p-value^a^** |
| --- | --- | --- | --- | --- |
|  | n = 113,354 | n = 1,089 | n = 114,443 |  |
| **DP1 z-score (SD)** | -0.011 (1.45) | 0.142 (1.45) | -0.009 (1.45) | **0.001** |
| **DP2 z-score (SD)** | 0.002 (1.04) | -0.058 (1.08) | 0.001 (1.04) | 0.06 |
| **Sex (n, %)** |  |  |  | <0.001 |
| Female | 62,992 (55.6%) | 462 (42.4%) | 63,454 (55.4%) |  |
| Male | 50,362 (44.4%) | 627 (57.6%) | 50,989 (44.6%) |  |
| **Age, years (SD)** | 55.9 (7.8) | 60.1 (6.7) | 55.9 (7.8) | **<0.001** |
| **BMI, kg/m2 (SD)** | 26.7 (4.6) | 27.5 (4.6) | 26.7 (4.6) | **<0.001** |
| **Obesity status (n, %)** |  |  |  | **<0.001** |
| Underweight | 647 (0.6%) | 5 (0.5%) | 652 (0.6%) |  |
| Healthy weight | 44,464 (39.2%) | 330 (30.3%) | 44,794 (39.1%) |  |
| Overweight | 46,253 (40.8%) | 495 (45.5%) | 46,748 (40.8%) |  |
| Obese | 21,990 (19.4%) | 259 (23.8%) | 22,249 (19.4%) |  |
| **Smoking status (n, %)** |  |  |  | **<0.001** |
| **Never** | 65,514 (57.8%) | 505 (46.4%) | 66,019 (57.7%) |  |
| **Previous** | 39,996 (35.3%) | 495 (45.5%) | 40,491 (35.4%) |  |
| **Current** | 7,844 (6.9%) | 89 (8.2%) | 7,933 (6.9%) |  |
| **TDI, quintiles (n, %)** |  |  |  | 0.49 |
| Quintile 1 | 22,735 (20.1%) | 223 (20.5%) | 22,958 (20.1%) |  |
| Quintile 2 | 22,719 (20.0%) | 239 (21.9%) | 22,958 (20.1%) |  |
| Quintile 3 | 22,708 (20.0%) | 217 (19.9%) | 22,925 (20.0%) |  |
| Quintile 4 | 22,685 (20.0%) | 204 (18.7%) | 22,889 (20.0%) |  |
| Quintile 5 | 22,507 (19.9%) | 206 (18.9%) | 22,713 (19.8%) |  |
| **Education (n, %)** |  |  |  | **<0.001** |
| Higher degree (college, university or professional degree/qualification) | 58,672 (51.8%) | 552 (50.7%) | 59,224 (51.7%) |  |
| Any school degree (A-level, AS-level, O-level, GCSE, CSE) | 33,215 (29.3%) | 324 (29.8%) | 33,539 (29.3%) |  |
| Vocational qualification (NVQ, HND or HNC) | 14,314 (12.6%) | 114 (10.5%) | 14,428 (12.6%) |  |
| None of the above | 7,153 (6.3%) | 99 (9.1%) | 7,252 (6.3%) |  |
| **Physical activity (n, %)** |  |  |  | **0.042** |
| Low | 22,054 (19.5%) | 226 (20.8%) | 22,280 (19.5%) |  |
| Moderate | 51,030 (45.0%) | 516 (47.4%) | 51,546 (45.0%) |  |
| High | 40,270 (35.5%) | 347 (31.9%) | 40,617 (35.5%) |  |
| **Diabetes diagnosis** | 4,211 (3.7%) | 76 (7.0%) | 4,287 (3.7%) | **<0.001** |
| **Family history of CRC** | 11,382 (10.0%) | 146 (13.4%) | 11,528 (10.1%) | **<0.001** |
| **Energy intake, kJ/day (SD)** | 8578.5 (2004.4) | 8832.4 (1925.0) | 8581.0 (2003.8) | **<0.001** |

^a^Two-tailed *t-*test, analysis of variance or Chi-squared test where appropriate. Abbreviations: CRC, colorectal cancer; SD, standard deviation; BMI, body mass index; TDI, Townsend deprivation index; Physical activity defined using International Physical Activity Questionnaire (IPAQ) metabolic equivalent (MET) scores: Low (<600 MET-minutes per week); Moderate (≥600 and <3000 MET-minutes per week); High (≥ 3000 MET-minutes per week). GCSE, general certificate of secondary education; CSE, certificate of secondary education; NVQ, national vocational qualification; HND, higher national diplomas; HNC, higher national certificate.
