# Supplementary material for: Associations between Dietary Patterns and Incident Colorectal Cancer in 114,443 Individuals from the UK Biobank: A Prospective Cohort Study
Source: Cancer Epidemiol Biomarkers Prev. 2024 Aug 19;33(11):1445–55. doi: 10.1158/1055-9965.EPI-24-0048 (PMC11528196; doi:10.1158/1055-9965.EPI-24-0048)
Supplement: Supplementary Table S4 — Table S4 Sequential adjustment of the HRs with 95% CIs for DP1’s association with incident CRC. [file epi-24-0048_supplementary_table_s4_suppst4.docx]

***Table S4:*** *Sequential adjustment of the HRs with 95% CIs for DP1’s association with incident CRC.*

| **Dietary pattern 1 variable form** | **No. events** | **Adjusted for age, sex** | **+Adjusted and stratified for behavioural factors^§^, family history of CRC** | **+Adjusted for SES*** | **+Adjusted for BMI, diabetes)** |
| --- | --- | --- | --- | --- | --- |
| **DP1 z-score linear form** | 1089 | **1.10**  **(1.06 - 1.15)** | **1.08**  **(1.03 - 1.12)** | **1.08**  **(1.03 - 1.13)** | **1.07**  **(1.03 - 1.12)** |
| **DP1 z-score quintiles** | | | | | |
| Quintile 1 | 180 | 1.00 (. - .) | 1.00 (. - .) | 1.00 (. - .) | 1.00 (. - .) |
| Quintile 2 | 216 | **1.22**  **(1.00 - 1.49)** | 1.21  (0.99 - 1.47) | 1.20  (0.99 - 1.47) | 1.18  (0.97 - 1.44) |
| Quintile 3 | 213 | **1.22**  **(1.00 - 1.49)** | 1.19  (0.98 - 1.46) | 1.19  (0.98 - 1.45) | 1.17  (0.96 - 1.43) |
| Quintile 4 | 238 | **1.38**  **(1.14 - 1.68)** | **1.31**  **(1.08 - 1.60)** | **1.31**  **(1.08 - 1.60)** | **1.29**  **(1.06 - 1.57)** |
| Quintile 5 | 242 | **1.51**  **(1.24 - 1.84)** | **1.37**  **(1.12 - 1.68)** | **1.37**  **(1.11 - 1.68)** | **1.34**  **(1.09 - 1.64)** |
| **LRT Chi-squared for DP1 z-scores, linear form^¶^** |  | *X*^2^ = 20.22,  d.f.(1),  **p < 0.0001** | *X*^2^ = 10.39,  d.f.(1),  **p = 0.0013** | *X*^2^ = 10.38,  d.f.(1)  **p =0.0013** | *X*^2^ = 9.43,  d.f.(1)  **p = 0.0026** |
| **LRT Chi-squared for trend across DP1 z-score quintiles^¶^** |  | *X*^2^ = 18.07,  d.f.(1),  **p < 0.0001** | *X*^2^ = 9.28,  d.f.(1),  **p = 0.0023** | *X*^2^ = 9.27,  d.f.(1),  **p = 0.0023** | *X*^2^ = 7.90,  d.f.(1),  **p = 0.005** |
| **LRT Chi-squared DP1 z-score quintiles^¶^** |  | *X*^2^ = 19.23,  d.f.(4),  **p = 0.0007** | *X*^2^ = 10.56,  d.f.(4),  **p = 0.0319** | *X*^2^ 10.51,  d.f.(4),  **p = 0.0326** | *X*^2^ = 9.17,  d.f.(4),  **p = 0.0569** |
| **DP1 z-score quintiles, floating absolute risk method** | | | | | |
| Quintile 1 | 180 | 1.00  (0.86 - 1.16) | 1.00  (0.86 - 1.16) | 1.00  (0.86 - 1.16) | 1.00  (0.86 - 1.16) |
| Quintile 2 | 216 | **1.22**  **(1.07 - 1.40)** | **1.21**  **(1.06 - 1.38)** | **1.18**  **(1.03 - 1.35)** | **1.18**  **(1.03 - 1.36)** |
| Quintile 3 | 213 | **1.22**  **(1.07 - 1.40)** | **1.19**  **(1.04 - 1.36)** | **1.17**  **(1.02 - 1.33)** | **1.17**  **(1.02 - 1.34)** |
| Quintile 4 | 238 | **1.38**  **(1.22 - 1.57)** | **1.31**  **(1.16 - 1.49)** | **1.31**  **(1.15 - 1.48)** | **1.29**  **(1.14 - 1.47)** |
| Quintile 5 | 242 | **1.51**  **(1.33 - 1.72)** | **1.37**  **(1.19 - 1.57)** | **1.36**  **(1.19 - 1.56)** | **1.34**  **(1.17 - 1.54)** |

Note: Adjusted hazard ratios (HR) and 95% confidence intervals (CI) of total DP1 z-scores (linear form) obtained using Cox proportional hazard regression. Adjusted HRs and 95% CIs of DP1 z-score quintiles were obtained using Cox proportional hazard regression (upper half of table). CIs obtained using the floating absolute risk method are presented in the bottom half of the table. **^§^**Behavioural risk factors: smoking status, total daily energy intake, physical activity level. ^*^Socioeconomic status (SES) proxies: Townsend deprivation index and educational attainment. Models were stratified by physical activity level, educational attainment, family history of CRC and BMI to address proportional hazards assumptions violations. **^¶^**Chi-squared values were calculated by likelihood ratio test, to measure the extent to which the dietary pattern is associated with incident overall CRC in the sequentially adjusted and stratified models (i.e. comparing each model with and without the dietary pattern) Abbreviations: CRC, colorectal cancer; DP1, dietary pattern 1; LRT, likelihood ratio test; BMI, body mass index; D.f., degrees of freedom.
