# Supplementary material for: Associations between Dietary Patterns and Incident Colorectal Cancer in 114,443 Individuals from the UK Biobank: A Prospective Cohort Study
Source: Cancer Epidemiol Biomarkers Prev. 2024 Aug 19;33(11):1445–55. doi: 10.1158/1055-9965.EPI-24-0048 (PMC11528196; doi:10.1158/1055-9965.EPI-24-0048)
Supplement: Supplementary Table S5 — Table S5 Hazard ratios and 95% confidence intervals of all variables in the fully-adjusted model for DP1 [file epi-24-0048_supplementary_table_s5_suppst5.docx]

***Table S5:*** *Hazard ratios and 95% confidence intervals of all variables in the fully-adjusted model for DP1*

| **Variables** | **HR (95% CI)** | **Standard error** | **p-value** |
| --- | --- | --- | --- |
| **Dietary pattern 1 z-scores, quintiles** |  |  |  |
| Quintile 1 | Reference | - | - |
| Quintile 2 | 1.18 (0.97 - 1.44) | 0.1008 | 0.09 |
| Quintile 3 | 1.17 (0.96 - 1.43) | 0.1017 | 0.12 |
| Quintile 4 | 1.29 (1.06 - 1.57) | 0.1002 | 0.01 |
| Quintile 5 | 1.34 (1.09 - 1.64) | 0.1042 | 0.006 |
| **Age, years** | 1.08 (1.07 - 1.09) | 0.0052 | < 0.001 |
| **Sex** |  |  |  |
| Female | Reference | - | - |
| Male | 1.34 (1.18 - 1.53) | 0.0901 | < 0.001 |
| **Smoking status** | |  |  |
| Never | Reference | - | - |
| Previous | 1.30 (1.15 – 1.48) | 0.0843 | < 0.001 |
| Current | 1.46 (1.16 – 1.84) | 0.1718 | 0.001 |
| **Total daily energy intake, log-kJ** | 1.38 (1.05 – 1.82) | 0.1947 | 0.02 |
| **TDI, quintiles** | |  |  |
| Quintile 1 | Reference | - | - |
| Quintile 2 | 1.04 (0.87 - 1.25) | 0.0969 | 0.6803 |
| Quintile 3 | 0.97 (0.81 - 1.17) | 0.0929 | 0.7630 |
| Quintile 4 | 0.93 (0.77 - 1.13) | 0.0906 | 0.4552 |
| Quintile 5 | 1.00 (0.82 - 1.21) | 0.0978 | 0.9789 |
| **Diagnosis of diabetes** | |  |  |
| **No** | Reference | - | - |
| **Yes** | 1.42 (1.12 – 1.80) | 0.1739 | 0.004 |

Note: 95% confidence intervals shown are *without* the floating absolute risk method applied. The fully-adjusted model was adjusted for age at baseline (not attained age at diagnosis or censoring), sex, smoking status, total daily energy intake (log-kJ), Townsend deprivation index (quintiles), and diabetes status. The model was also stratified by BMI (underweight, healthy weight, overweight, obese), physical activity level (MET-hours per week : low, moderate, high), educational attainment (higher degree, any school degree, vocational qualification, none of the above) and family history of CRC. Abbreviations: HR, hazard ratio; 95% CI, 95% confidence interval; TDI, Townsend deprivation index.
