# Supplementary material for: Associations between Dietary Patterns and Incident Colorectal Cancer in 114,443 Individuals from the UK Biobank: A Prospective Cohort Study
Source: Cancer Epidemiol Biomarkers Prev. 2024 Aug 19;33(11):1445–55. doi: 10.1158/1055-9965.EPI-24-0048 (PMC11528196; doi:10.1158/1055-9965.EPI-24-0048)
Supplement: Supplementary Table S6 — Table S6 Subgroup analysis of the association between DP1 and incident CRC by anatomic subgroup, fully adjusted and stratified model. [file epi-24-0048_supplementary_table_s6_suppst6.docx]

***Table S6:*** *Subgroup analysis of the association between DP1 and incident CRC by anatomic subgroup, fully adjusted and stratified model.*

|  | **All CRC cases** | **Colon cancers^*^** | **Proximal colon cancers** | **Distal colon cancers** | **Rectal cancers** |
| --- | --- | --- | --- | --- | --- |
| **Total participants, n** | 114,443 | 114,443 | 114,443 | 114,443 | 114,443 |
| **Cases, n** | 1,089 | 637 | 396 | 241 | 380 |
| **DP1 z-scores, linear form** | **1.07**  **(1.03 - 1.12)** | 1.04  (0.98 - 1.10) | 1.03  (0.96 - 1.11) | 1.05  (0.96 - 1.15) | **1.14**  **(1.05 - 1.23)** |
| **DP z-score quintiles** | |  |  |  |  |
| Quintile 1 | 1.00 (. - .) | 1.00 (. - .) | 1.00 (. - .) | 1.00 (. - .) | 1.00 (. - .) |
| Quintile 2 | 1.18  (0.97 - 1.44) | 1.14  (0.88 - 1.47) | 1.21  (0.89 - 1.66) | 1.01  (0.65 - 1.58) | 1.09  (0.77 - 1.55) |
| Quintile 3 | 1.17  (0.96 - 1.43) | 1.09  (0.84 - 1.41) | 1.09  (0.79 - 1.52) | 1.08  (0.70 - 1.67) | 1.30  (0.93 - 1.82 |
| Quintile 4 | **1.29**  **(1.06 - 1.57)** | **1.33**  **(1.03 - 1.71)** | 1.34  (0.98 - 1.84) | 1.32  (0.87 - 2.01) | 1.23  (0.87 - 1.73) |
| Quintile 5 | **1.34**  **(1.09 - 1.64)** | 1.20  (0.91 -1.57) | 1.11  (0.78 - 1.57) | 1.32  (0.85 - 2.03) | **1.58**  **(1.12 - 2.23)** |
| **LRT Chi-squared for DP1 z-scores, linear form^¶^** | ***X*^2^=9.43, d.f.(1),**  **p =0.0026** | *X*^2^ = 1.71, d.f.(1),  p = 0.191 | *X*^2^=0.67, d.f.(1),  p = 0.412 | *X*^2^=1.07, d.f.(1),  p = 0.301 | ***X*^2^=10.53, d.f.(1),**  **p = 0.0012** |
| **Test for trend across DP z-score quintiles^¶^** | ***X*^2^=7.90, d.f.(1), p = 0.005** | *X*^2^ = 2.94, d.f.(1), p = 0.086 | *X*^2^=0.74, d.f.(1),  p = 0.389 | *X*^2^=2.78, d.f.(1),  p = 0.096 | ***X*^2^ = 6.82, d.f.(1),**  **p = 0.009** |
| **DP1 z-score quintiles, floating absolute risk method** | | | | | |
| Quintile 1 | 1.00  (0.86 - 1.16) | 1.00  (0.82 - 1.21) | 1.00  (0.79 - 1.27) | 1.00  (0.72 - 1.39) | 1.00  (0.77 - 1.29) |
| Quintile 2 | **1.18**  **(1.03 - 1.36)** | 1.14  (0.95 - 1.36) | 1.21  (0.98 - 1.50) | 1.01  (0.74 - 1.39) | 1.09  (0.86 -1.39) |
| Quintile 3 | **1.17**  **(1.02 - 1.34)** | 1.09  (0.91 - 1.30) | 1.09  (0.87 - 1.37) | 1.08  (0.80 - 1.45) | **1.30**  **(1.04 - 1.62)** |
| Quintile 4 | **1.29**  **(1.14 - 1.47)** | **1.33**  **(1.13 - 1.56)** | **1.34**  **(1.09 - 1.64)** | **1.32**  **(1.02 - 1.71)** | 1.23  (0.98 - 1.53) |
| Quintile 5 | **1.34**  **(1.16 - 1.53)** | 1.20  (0.99 - 1.44) | 1.11  (0.86 -1.42) | **1.32**  **(1.00 - 1.73)** | **1.58**  **(1.27 - 1.96)** |

Adjusted hazard ratios (HR) and 95% confidence intervals (CI) of total DP z-scores (linear form) obtained using Cox proportional hazard regression. Adjusted HRs and 95% CIs of DP1 z-score quintiles were obtained using Cox proportional hazard regression (upper half of table). CIs obtained using the floating absolute risk method are presented in the bottom half of the table. The model used was adjusted for age at baseline (not attained age at diagnosis or censoring), sex, smoking status, total daily energy intake (log-kJ), Townsend deprivation index (quintiles), and diabetes status. The model was also stratified by BMI (underweight, healthy weight, overweight, obese), physical activity level (MET-hours per week : low, moderate, vigorous), educational attainment (higher degree, any school degree, vocational qualification, none of the above) and family history of CRC. *Unspecified and overlapping colorectal cancers were excluded from the analysis since they could not be precisely localized to an anatomic subsite. **^¶^**Chi-squared values were calculated by likelihood ratio test, to measure the extent to which the dietary pattern is associated with incident overall CRC in the sequentially adjusted and stratified models (i.e. comparing each model with and without the dietary pattern). Abbreviations: CRC, colorectal cancer; DP, dietary pattern; LRT, likelihood ratio test; *X^2^,* chi-squared.
