# Supplementary material for: Associations between Dietary Patterns and Incident Colorectal Cancer in 114,443 Individuals from the UK Biobank: A Prospective Cohort Study
Source: Cancer Epidemiol Biomarkers Prev. 2024 Aug 19;33(11):1445–55. doi: 10.1158/1055-9965.EPI-24-0048 (PMC11528196; doi:10.1158/1055-9965.EPI-24-0048)
Supplement: Supplementary Table S8 — Table S8 Hazard ratios and 95% confidence intervals of all variables in the fully-adjusted model for DP2 [file epi-24-0048_supplementary_table_s8_suppst8.docx]

***Table S8:*** *Hazard ratios and 95% confidence intervals of all variables in the fully-adjusted model for DP2*

| **Variables** | **HR (95% CI)** | **Standard error** | **p-value** |
| --- | --- | --- | --- |
| **Dietary pattern 2 z-scores, quintiles** |  |  |  |
| Quintile 1 | Reference | - | - |
| Quintile 2 | 0.87 (0.72 - 1.05) | 0.0828 | 0.140 |
| Quintile 3 | 0.90 (0.75 - 1.08) | 0.0853 | 0.267 |
| Quintile 4 | 0.86 (0.71 - 1.04) | 0.0825 | 0.112 |
| Quintile 5 | 0.94 (0.78 - 1.12) | 0.0877 | 0.48 |
| **Age, years** | 1.08 (1.07 - 1.09) | 0.0051 | <0.001 |
| **Sex** |  |  |  |
| Female | Reference | - | - |
| Male | 1.4 (1.23 - 1.59) | 0.0923 | <0.001 |
| **Smoking status** |  |  |  |
| Never | Reference | - | - |
| Previous | 1.30 (1.15 - 1.48) | 0.0847 | <0.001 |
| Current | 1.50 (1.20 - 1.89) | 0.1779 | <0.001 |
| **Total daily energy intake, log-kJ** | 1.43 (1.09 - 1.89) | 0.1971 | 0.01 |
| **TDI, quintiles** |  |  |  |
| Quintile 1 | Reference |  |  |
| Quintile 2 | 1.06 (0.88 - 1.27) | 0.0989 | 0.530 |
| Quintile 3 | 0.98 (0.81 - 1.18) | 0.0938 | 0.842 |
| Quintile 4 | 0.96 (0.79 - 1.16) | 0.0931 | 0.640 |
| Quintile 5 | 1.02 (0.84 - 1.24) | 0.1000 | 0.837 |
| **Diagnosis of diabetes** |  |  |  |
| No | Reference | - | - |
| Yes | 1.37 (1.09 – 1.77) | 0.1708 | 0.007 |

Note: The fully-adjusted model was adjusted for age at baseline (not attained age at diagnosis or censoring), sex, smoking status, total daily energy intake (log-kJ), Townsend deprivation index (quintiles), and diabetes status. The model was also stratified by BMI (underweight, healthy weight, overweight, obese), physical activity level (MET-hours per week : low, moderate, high), educational attainment (higher degree, any school degree, vocational qualification, none of the above) and family history of CRC. Abbreviations: HR, hazard ratio; 95% CI, 95% confidence interval; TDI, Townsend deprivation index.
