# Supplementary material for: Associations between Dietary Patterns and Incident Colorectal Cancer in 114,443 Individuals from the UK Biobank: A Prospective Cohort Study
Source: Cancer Epidemiol Biomarkers Prev. 2024 Aug 19;33(11):1445–55. doi: 10.1158/1055-9965.EPI-24-0048 (PMC11528196; doi:10.1158/1055-9965.EPI-24-0048)
Supplement: Supplementary Table S9 — Table S9 Sensitivity analyses of the fully adjusted and stratified model for the association between dietary pattern 1 and incident overall CRC [file epi-24-0048_supplementary_table_s9_suppst9.docx]

***Table S9:*** *Sensitivity analyses of the fully adjusted and stratified model for the association between dietary pattern 1 and incident overall CRC.*

|  | **Original model** | **Prior lower endoscopy** | **Alternate measure of adiposity (WC)** | **3+ WebQs completed** | **No major dietary change in last 5 years** | **Excluding current/ever smokers** | **Excluding first 3 years after WebQ completion** |
| --- | --- | --- | --- | --- | --- | --- | --- |
| **Total participants, n** | 114,443 | 102,415 | 114,443 | 71,184 | 72,590 | 66,019 | 114,141 |
| **Cases, n** | 1089 | 986 | 1,089 | 663 | 674 | 505 | 787 |
| **HR, 95% CI for DP1 z-scores, linear form** | **1.07**  **(1.03 - 1.12)** | **1.07**  **(1.02 - 1.12)** | **1.07**  **(1.02 - 1.12)** | 1.03  (0.97 - 1.10) | **1.11**  **(1.05 - 1.18)** | **1.10**  **(1.03 - 1.18)** | **1.08**  **(1.03 - 1.14)** |
| **HR, 95% CI for DP1 z-score quintiles** | | | | | | | |
| Quintile 1 | 1.00 (. - .) | 1.00 (. - .) | 1.00 (. - .) | 1.00 (. - .) | 1.00 (. - .) | 1.00 (. - .) | 1.00 (. - .) |
| Quintile 2 | 1.18  (0.97 - 1.44) | 1.15  (0.93 - 1.42) | 1.18  (0.97 - 1.45) | 1.15  (0.88 - 1.47) | 1.13  (0.87 - 1.48) | **1.38**  **(1.04 - 1.85)** | 1.19  (0.94 - 1.51) |
| Quintile 3 | 1.17  (0.96 - 1.43) | 1.15  (0.93 - 1.41) | 1.15  (0.94 - 1.41) | 1.18  (0.90 - 1.50) | 1.07  (0.82 - 1.40) | **1.41**  **(1.05 - 1.88)** | 1.25  (0.99 - 1.58) |
| Quintile 4 | **1.29**  **(1.06 - 1.57)** | **1.25**  **(1.02 - 1.54)** | **1.27**  **(1.04 - 1.56)** | **1.32**  **(1.02 - 1.69)** | **1.42**  **(1.10 - 1.83)** | **1.60**  **(1.20 – 2.14)** | **1.34**  **(1.06 - 1.69)** |
| Quintile 5 | **1.34**  **(1.09 - 1.64)** | **1.37**  **(1.10 - 1.69)** | **1.32**  **(1.07 - 1.63)** | 1.09  (0.82 - 1.43) | **1.51**  **(1.16 - 1.96)** | **1.50**  **(1.09 - 2.05)** | **1.36**  **(1.07 - 1.74)** |
| **LRT Chi-squared for DP1 z-scores, linear form^a^** | *X^2^=*9.05, d.f.(1),  **p = 0.003** | *X^2^*= 7.97, d.f.(1),  **p = 0.005** | *X^2^*= 8.31, d.f. (1),  **p = 0.003** | *X^2^*= 1.14, d.f.(1),  p = 0.29 | *X^2^*= 11.76, d.f.(1),  **p < 0.001** | *X^2^*= 8.03,d.f.(1),  **p = 0.005** | *X^2^*= 8.11, d.f.(1),  **p = 0.004** |
| **Test for trend across DP1 z-score quintiles^a^** | *X^2^*= 7.90, d.f.(1),  **p= 0.005** | *X^2^* = 7.94, d.f.(1),  **p = 0.005** | *X^2^*= 6.77, d.f.(1),  **p = 0.009** | *X^2^*= 1.38, d.f.(1),  p = 0.24 | *X^2^*= 11.89, d.f.(1),  **p < 0.001** | *X^2^*= 7.42, d.f.(1),  **p = 0.007** | *X^2^* = 6.51, d.f.(1),  **p = 0.01** |
| **HR, 95% CI DP1 quintiles - floating absolute risk method for confidence intervals** | | | | | | | |
| Quintile 1 | 1.00  (0.86 - 1.16) | 1.00  (0.85 - 1.17) | 1.00  (0.86 - 1.17) | 1.00  (0.82 - 1.21) | 1.00  (0.82 - 1.22) | 1.00  (0.80 - 1.25) | 1.00  (0.84 - 1.20) |
| Quintile 2 | **1.18**  **(1.03 - 1.36)** | 1.15  (0.99 - 1.33) | **1.18**  **(1.03 - 1.36)** | 1.15  (0.98 - 1.38) | 1.13  (0.95 - 1.36) | **1.38**  **(1.14 - 1.68)** | **1.19**  **(1.02 - 1.40)** |
| Quintile 3 | **1.17**  **(1.02 - 1.34)** | 1.15  (0.99 - 1.32) | **1.15**  **(1.00 - 1.32)** | **1.18**  **(1.00 - 1.39)** | 1.07  (0.89 - 1.28) | **1.41**  **(1.17 - 1.70)** | **1.25**  **(1.07 - 1.46)** |
| Quintile 4 | **1.29**  **(1.14 - 1.47)** | **1.25**  **(1.09 - 1.43)** | **1.27**  **(1.12 - 1.45)** | **1.32**  **(1.14 - 1.55)** | **1.42**  **(1.22 - 1.65)** | **1.60**  **(1.33 - 1.93)** | **1.34**  **(1.15 - 1.56)** |
| Quintile 5 | **1.34**  **(1.17 - 1.54)** | **1.37**  **(1.18 - 1.58)** | **1.31**  **(1.14 - 1.51)** | 1.09  (0.90 - 1.31) | **1.51**  **(1.28 - 1.78)** | **1.50**  **(1.20 - 1.86)** | **1.36**  **(1.16 - 1.60)** |

The fully adjusted model was adjusted for age at baseline (not attained age at diagnosis or censoring), sex, smoking status, total daily energy intake (log-kJ), Townsend deprivation index (quintiles), and diabetes status. The model was also stratified by BMI (underweight, healthy weight, overweight, obese), physical activity level (MET-hours per week : low, moderate, vigorous), educational attainment (higher degree, any school degree, vocational qualification, none of the above) and family history of CRC. **^a^**Chi-squared values were calculated by likelihood ratio test, to measure the extent to which the dietary pattern is associated with incident overall CRC in the sequentially adjusted and stratified models (i.e. comparing each model with and without the dietary pattern). Abbreviations: DP1, dietary pattern 1; HR, hazard ratio; 95% CI, 95% confidence interval; WC, waist circumference.
