# Supplementary material for: Associations between Dietary Patterns and Incident Colorectal Cancer in 114,443 Individuals from the UK Biobank: A Prospective Cohort Study
Source: Cancer Epidemiol Biomarkers Prev. 2024 Aug 19;33(11):1445–55. doi: 10.1158/1055-9965.EPI-24-0048 (PMC11528196; doi:10.1158/1055-9965.EPI-24-0048)
Supplement: Supplementary Table S11 — Table S11. Comparison of incident colorectal cancer cases and baseline characteristics between those completing 2 vs. 3+ WebQs [file epi-24-0048_supplementary_table_s11_suppst11.docx]

***Table S11****.* *Comparison of incident colorectal cancer cases and baseline characteristics between those completing 2 vs. 3+ WebQs*

|  | **2 WebQs only** | **3+ WebQs** | **Total** |
| --- | --- | --- | --- |
|  | N=43,259 | N=71,184 | N=114,443 |
| **Colorectal cancer cases (n, %)** | 426 (1.0%) | 663 (0.9%) | 1,089 (1.0%) |
| **Sex (n, %)** |  |  |  |
| Female | 23,834 (55.1%) | 39,620 (55.7%) | 63,454 (55.4%) |
| Male | 19,425 (44.9%) | 31,564 (44.3%) | 50,989 (44.6%) |
| **Age, years (SD)** | 55.6 (8.0) | 56.1 (7.7) | 55.9 (7.8) |
| **BMI, kg/m^2^ (SD)** | 26.9 (4.6) | 26.6 (4.6) | 26.7 (4.6) |
| **Smoking status (n, %)** |  |  |  |
| Never | 24,654 (57.0%) | 41,365 (58.1%) | 66,019 (57.7%) |
| Previous | 15,301 (35.4%) | 25,190 (35.4%) | 40,491 (35.4%) |
| Current | 3,304 (7.6%) | 4,629 (6.5%) | 7,933 (6.9%) |
| **Townsend deprivation index, quintiles (n, %)** |  |  |  |
| Q1 | 8,801 (20.3%) | 14,157 (19.9%) | 22,958 (20.1%) |
| Q2 | 8,655 (20.0%) | 14,303 (20.1%) | 22,958 (20.1%) |
| Q3 | 8,836 (20.4%) | 14,089 (19.8%) | 22,925 (20.0%) |
| Q4 | 8,495 (19.6%) | 14,394 (20.2%) | 22,889 (20.0%) |
| Q5 | 8,472 (19.6%) | 14,241 (20.0%) | 22,713 (19.8%) |
| **Educational attainment (n, %)** |  |  |  |
| Higher degree | 21,660 (50.1%) | 37,564 (52.8%) | 59,224 (51.7%) |
| Any school degree | 12,742 (29.5%) | 20,797 (29.2%) | 33,539 (29.3%) |
| Vocational qualification | 5,779 (13.4%) | 8,649 (12.2%) | 14,428 (12.6%) |
| None of the above | 3,078 (7.1%) | 4,174 (5.9%) | 7,252 (6.3%) |
| **Physical Activity group (n, %)** |  |  |  |
| Low | 8,551 (19.8%) | 13,729 (19.3%) | 22,280 (19.5%) |
| Moderate | 19,263 (44.5%) | 32,283 (45.4%) | 51,546 (45.0%) |
| High | 15,445 (35.7%) | 25,172 (35.4%) | 40,617 (35.5%) |
| **Diabetes diagnosis (n, %)** | 1,695 (3.9%) | 2,592 (3.6%) | 4,287 (3.7%) |
| **Family history of CRC (n, %)** | 4,359 (10.1%) | 7,169 (10.1%) | 11,528 (10.1%) |
| **History of previous endoscopy (n, %)** | 4,668 (10.8%) | 7,360 (10.3%) | 12,028 (10.5%) |
| **DP1 quantiles (n, %)** |  |  |  |
| Q1 | 8,952 (20.7%) | 13,864 (19.5%) | 22,816 (19.9%) |
| Q2 | 8,329 (19.3%) | 14,619 (20.5%) | 22,948 (20.1%) |
| Q3 | 8,259 (19.1%) | 14,765 (20.7%) | 23,024 (20.1%) |
| Q4 | 8,502 (19.7%) | 14,575 (20.5%) | 23,077 (20.2%) |
| Q5 | 9,217 (21.3%) | 13,361 (18.8%) | 22,578 (19.7%) |
| **DP2 quantiles (n, %)** |  |  |  |
| Q1 | 8,849 (20.5%) | 13,863 (19.5%) | 22,712 (19.8%) |
| Q2 | 8,221 (19.0%) | 14,732 (20.7%) | 22,953 (20.1%) |
| Q3 | 8,202 (19.0%) | 14,821 (20.8%) | 23,023 (20.1%) |
| Q4 | 8,470 (19.6%) | 14,461 (20.3%) | 22,931 (20.0%) |
| Q5 | 9,517 (22.0%) | 13,307 (18.7%) | 22,824 (19.9%) |
| **Major diet change in last 5 years (n, %)** | 16,136 (37.3%) | 25,717 (36.1%) | 41,853 (36.6%) |
| **Energy intake, kJ/day (median, IQR)** | 8,388  (7,061 – 9,902) | 8,435  (7,250 – 12,057) | 8,418  (7,181 – 9,820) |

Higher degree defined as: college, university, or professional degree/qualification. Any school degree defined as: A-level, AS-level, O-level, GCSE (general certificate of secondary education) or CSE (certificate of secondary education). Vocational qualifications defined as: NVQ (national vocational qualification), HND (higher national diplomas), HNC (higher national certificate). Physical activity defined using International Physical Activity Questionnaire (IPAQ) metabolic equivalent (MET) scores: Low (<600 MET-minutes per week); Moderate (≥600 and <3000 MET-minutes per week); High (≥ 3000 MET-minutes per week). Abbreviations: SD, standard deviation; IQR, interquartile range; BMI, body mass index; CRC, colorectal cancer
