# Supplementary material for: Associations between Dietary Patterns and Incident Colorectal Cancer in 114,443 Individuals from the UK Biobank: A Prospective Cohort Study
Source: Cancer Epidemiol Biomarkers Prev. 2024 Aug 19;33(11):1445–55. doi: 10.1158/1055-9965.EPI-24-0048 (PMC11528196; doi:10.1158/1055-9965.EPI-24-0048)
Supplement: Supplementary Figure S1 — Figure S1 Directed acyclic graph of variables used in the model. [file epi-24-0048_supplementary_figure_s1_suppsf1.docx]

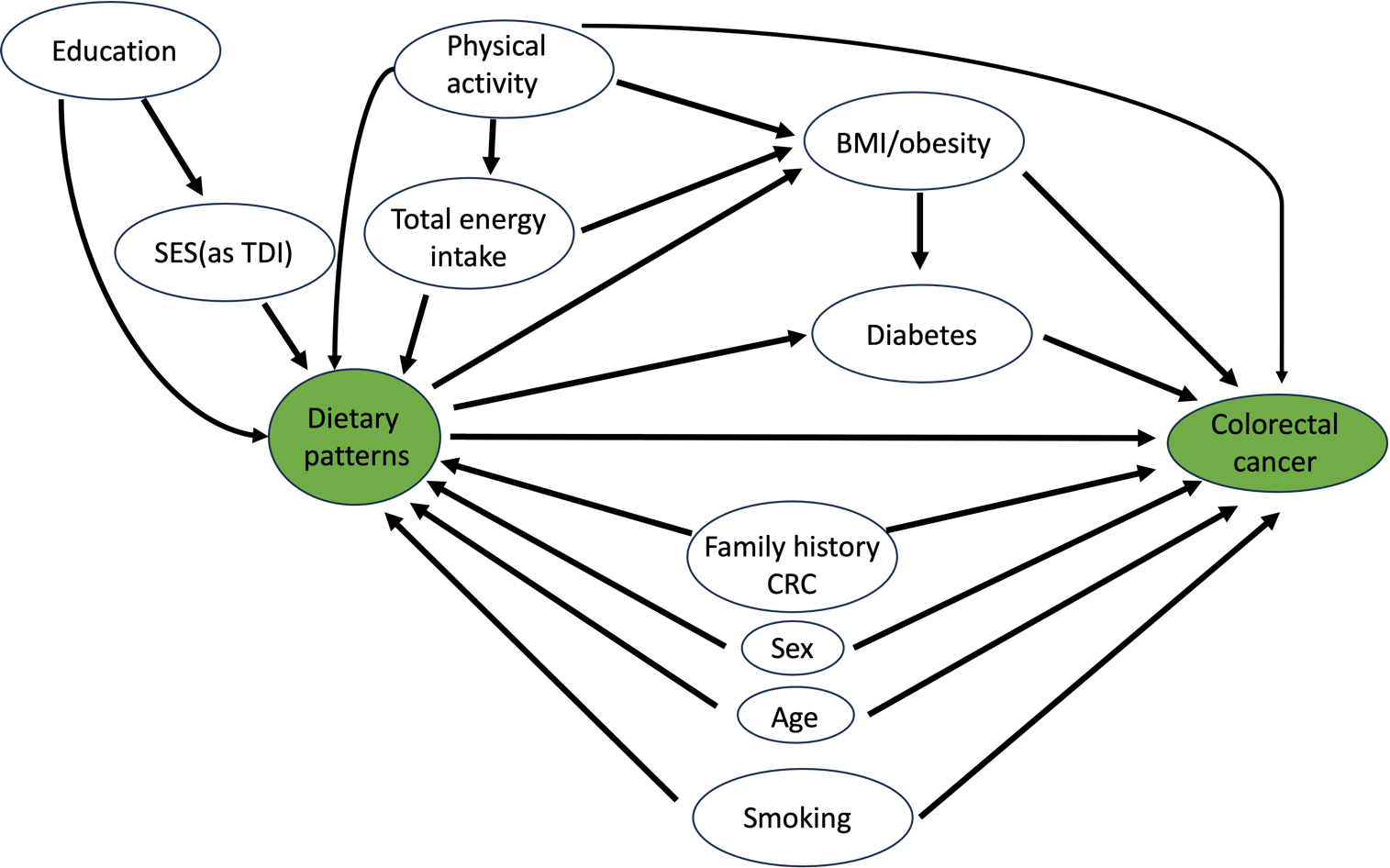


***Figure S1:*** *Directed acyclic graph of variables used in the model.* Abbreviations: BMI, body mass index; CRC, colorectal cancer; TDI, Townsend deprivation index; SES, socioeconomic status.
