# Supplementary material for: Associations between Dietary Patterns and Incident Colorectal Cancer in 114,443 Individuals from the UK Biobank: A Prospective Cohort Study
Source: Cancer Epidemiol Biomarkers Prev. 2024 Aug 19;33(11):1445–55. doi: 10.1158/1055-9965.EPI-24-0048 (PMC11528196; doi:10.1158/1055-9965.EPI-24-0048)
Supplement: Supplementary Figure S2 — Figure S2 U.K. Biobank participant exclusion flow chart [file epi-24-0048_supplementary_figure_s2_suppsf2.docx]

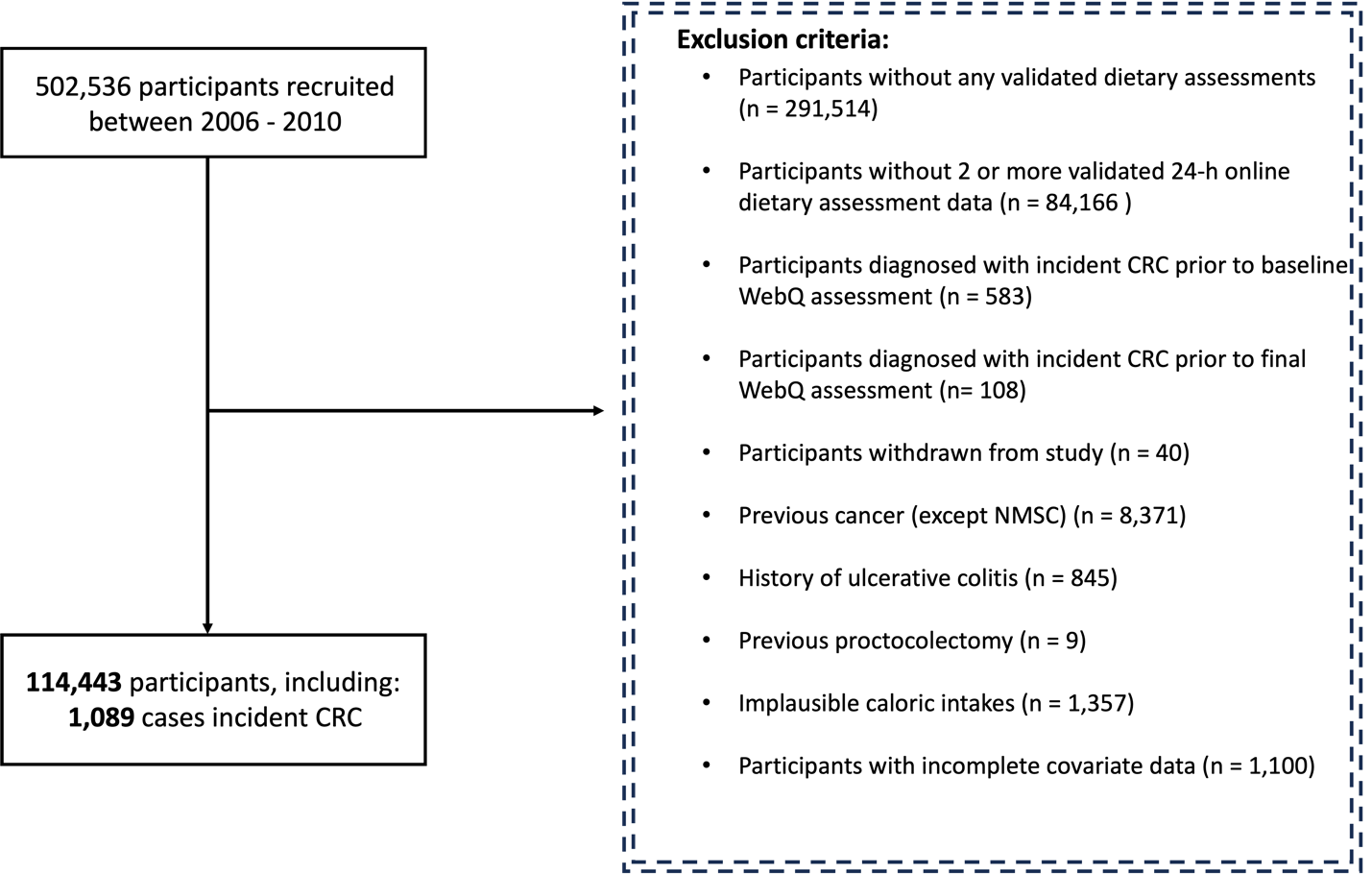


**Figure S2:** U.K. Biobank participant exclusion flow chart

CRC, colorectal cancer; NMSC, Nonmelanoma skin cancer; WebQ; Oxford WebQ 24-hour recall dietary tool
