# Supplementary material for: Associations between Dietary Patterns and Incident Colorectal Cancer in 114,443 Individuals from the UK Biobank: A Prospective Cohort Study
Source: Cancer Epidemiol Biomarkers Prev. 2024 Aug 19;33(11):1445–55. doi: 10.1158/1055-9965.EPI-24-0048 (PMC11528196; doi:10.1158/1055-9965.EPI-24-0048)
Supplement: Supplementary Figure S3 — Figure S3 Hazard ratios and 95% confidence intervals for interactions of DP1 and DP2 with age and sex [file epi-24-0048_supplementary_figure_s3_suppsf3.docx]

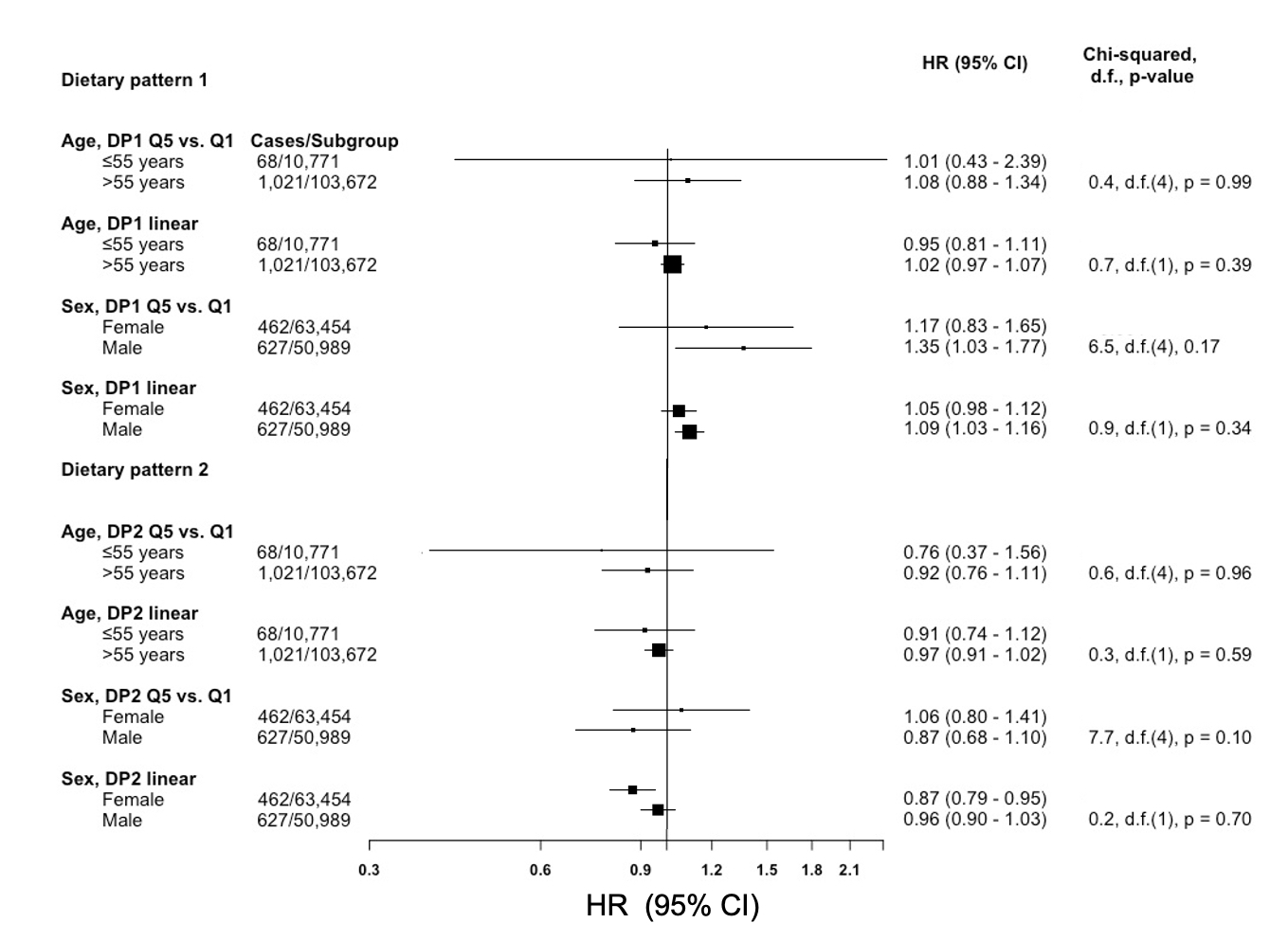


***Figure S3:*** *Hazard ratios and 95% confidence intervals for interactions of DP1 and DP2 with age and sex.* Likelihood ratio tests for interactions of age and sex with the dietary pattern z-scores in linear and quintile form. Note: X-axis represents HRs with associated 95% confidence intervals and is on the log scale. HRs shown for DP z-scores in linear form and for quintile 5 vs. 1. Right-hand column denotes the likelihood ratio test results for interaction. The fully adjusted model was adjusted for age (for the age interaction, age attained during follow-up was used and not age at baseline), sex, smoking status, total daily energy intake (log-kJ), Townsend deprivation index (quintiles), and diabetes status. The model was also stratified by BMI (underweight, healthy weight, overweight, obese), physical activity level (MET-hours per week : low, moderate, vigorous), educational attainment (higher degree, any school degree, vocational qualification, none of the above) and family history of CRC. DP, dietary pattern; Q1, quintile 1; Q5, quintile 5.
