# Supplementary material for: Associations between Dietary Patterns and Incident Colorectal Cancer in 114,443 Individuals from the UK Biobank: A Prospective Cohort Study
Source: Cancer Epidemiol Biomarkers Prev. 2024 Aug 19;33(11):1445–55. doi: 10.1158/1055-9965.EPI-24-0048 (PMC11528196; doi:10.1158/1055-9965.EPI-24-0048)
Supplement: Supplementary Figure S4 — Figure S4 DP2 associations with CRC by anatomic subsite [file epi-24-0048_supplementary_figure_s4_suppsf4.docx]

***
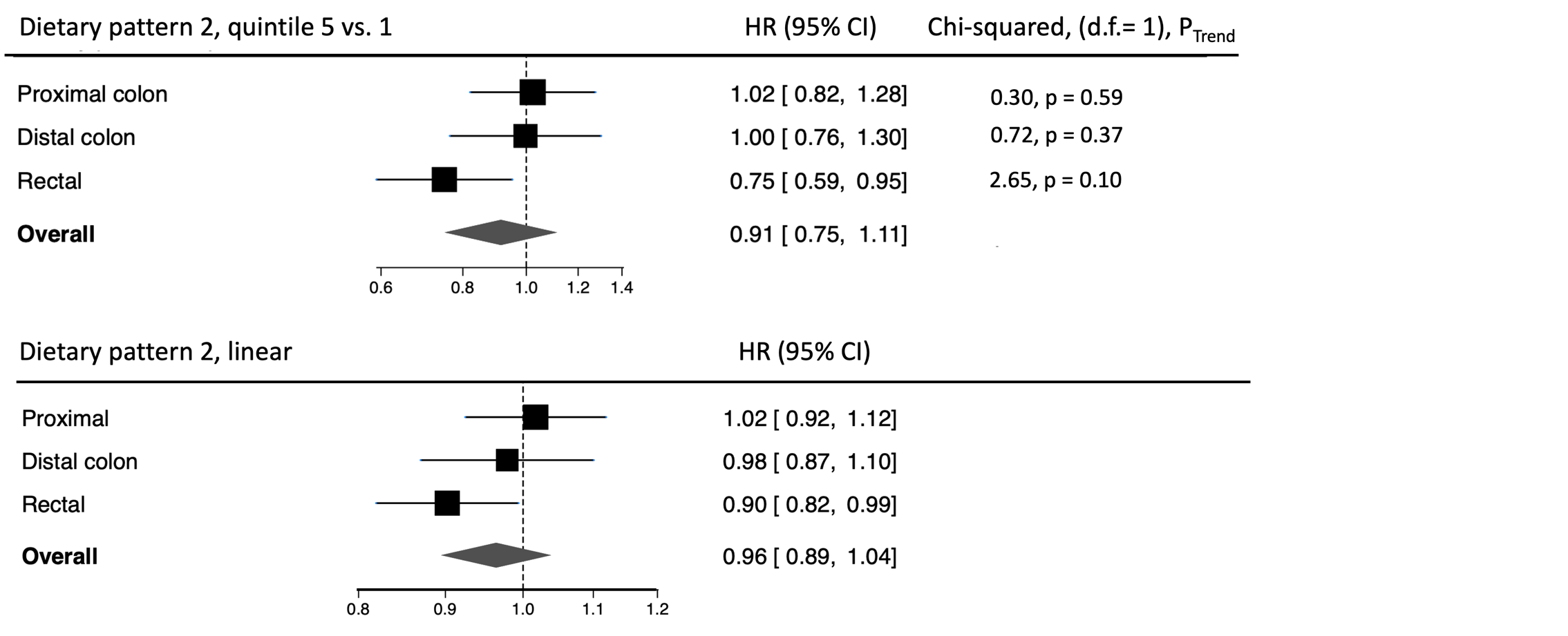
***

***Figure S4:*** *DP2 associations with CRC by anatomic subsite.* Top panel illustrates the HRs of quintile 5 vs. quintile 1 for each anatomic subsite. Bottom panel illustrates the HRs of DP2 per 1-SD increase. Unspecified (n = 68) and overlapping (n = 4) colorectal cancers were excluded as they could not be localized to an anatomic subsite. D.f., degrees of freedom.
