# Supplementary material for: Associations between Dietary Patterns and Incident Colorectal Cancer in 114,443 Individuals from the UK Biobank: A Prospective Cohort Study
Source: Cancer Epidemiol Biomarkers Prev. 2024 Aug 19;33(11):1445–55. doi: 10.1158/1055-9965.EPI-24-0048 (PMC11528196; doi:10.1158/1055-9965.EPI-24-0048)
Supplement: Supplementary Figure S5 — Figure S5 DP2 Sensitivity analyses [file epi-24-0048_supplementary_figure_s5_suppsf5.docx]

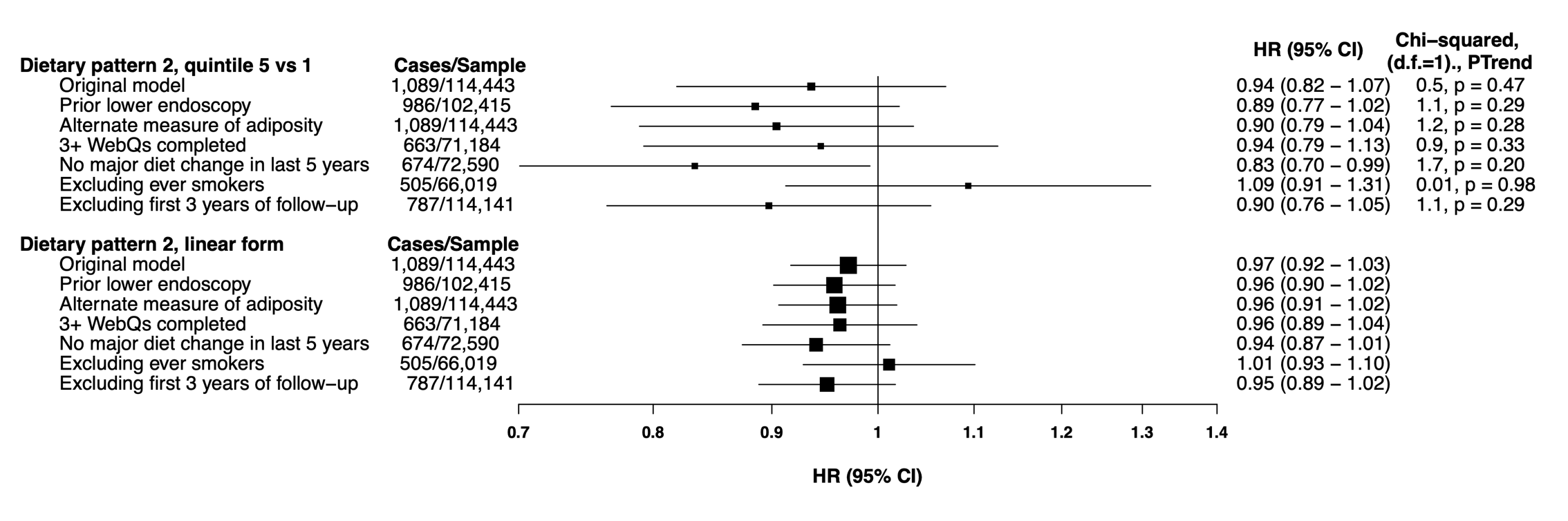


***Figure S5.*** *DP2 Sensitivity analyses.* The x-axis comprises hazard ratios and associated 95% confidence intervals and is on the log-scale. Abbreviations*:* DP, dietary pattern; WC, waist circumference; WebQ, Oxford WebQ 24-h dietary assessment tool. Confidence intervals presented were obtained using the floating absolute risk method.
